# Visual exposure to masked faces benefits personally familiar but not famous face recognition

**DOI:** 10.3389/fpsyg.2026.1671509

**Published:** 2026-04-17

**Authors:** Srijita Karmakar, Subhajit Das, Koel Das

**Affiliations:** 1 Department of Psychological and Brain Sciences, University of California Santa Barbara, Santa Barbara, CA, United States; 2 Indian Institute of Science Education and Research Kolkata, Kolkata, West Bengal, India

**Keywords:** EEG, face familiarity, face masks, face recognition, N170, N250, COVID-19

## Abstract

**Introduction:**

The introduction of face masks during the recent COVID-19 pandemic presented a potential challenge for human face perception and recognition. Our exploratory study investigates the effect of face masks in face recognition by probing the neuropsychological mechanisms of the same. We also aim to explicate the effect of general exposure to masked faces and visual experience with specific masked faces of personally familiar individuals on face recognition ability.

**Methods:**

Participants detected personally familiar, famous, and unfamiliar Indian faces in masked and unmasked conditions in a 2-back test.

**Results:**

Statistical analyses revealed significant main effects of familiarity and mask conditions on performance accuracy and reaction time (RT). The highest performance accuracy in correctly detecting the target face was observed for familiar and unmasked faces, and the lowest for unfamiliar and masked ones. Notably, RTs were not different between unmasked and masked personally familiar faces, while masked famous faces elicited significantly greater RT than their unmasked counterpart. EEG analysis was consistent with behavioral results. Specifically, we show neural evidence for increased effort in processing masked famous faces that is similar to that for masked unfamiliar faces, but absent for masked familiar faces. This increased effort to process masked faces of unfamiliar or famous individuals is suggestive of a beneficial effect of visual exposure to, and experience with, specific masked faces.

**Conclusion:**

In sum, our study presents behavioral and neural evidence that personal familiarity with masked faces aids perceptual learning and eventual recognition, and this learning does not generalize to otherwise well-known faces.

## Introduction

If the eyes are the windows to one's soul, the face may be considered a door open wide. Humans are highly efficient at recognizing faces, exhibiting robustness to low-level visual changes such as size, viewpoint, illumination, and context (see Johnston and Edmonds, 2009; Ramon and Gobbini, 2018 for reviews). Face recognition plays a central role in social interaction and communication (Avery et al., 2016), and recognition of personally familiar faces is markedly superior to that of unfamiliar faces (Bruce, 1994; Bruce et al., 2001; Burton et al., 2015; Ramon and Van Belle, 2016; Gobbini and Haxby, 2007).

The COVID-19 pandemic introduced a major visuo-social shift in early 2020 through the widespread adoption of face masks, which became mandatory in many countries. Since then, face masks have been rapidly integrated into daily life, substantially altering human social interaction. Given the importance of faces in communication, it is natural to ask how face masks, which occlude nearly 50% of the face, affect face perception, recognition, and broader social communication (Molnar-Szakacs et al., 2021). Face masks may negatively impact the typical face-recognition machinery of the human brain (Ferrari et al., 2021), a hypothesis supported by a growing body of experimental work conducted during the pandemic (Carragher and Hancock, 2020; Noyes et al., 2021; Gori et al., 2021; Carbon, 2020; Freud et al., 2020; Schroeter et al., 2021).

Behavioral evidence consistently shows that face masks impair face recognition. Using an adapted version of the Cambridge Face Memory Test (Duchaine and Nakayama, 2005), Freud et al. (2020) demonstrated that participants showed poorer memory for masked compared to unmasked faces. In addition to identity recognition, face masks also impair emotion and expression perception. Several studies report a detrimental effect of masks on emotion recognition accuracy (Gori et al., 2021; Carbon, 2020). Specifically, Carbon (2020) observed distinct confusion patterns in emotion judgments for masked faces, with many emotions misclassified as neutral, whereas Gori et al. (2021) found that emotion inference was particularly impaired in children, whose face-processing systems are still developing. Face masks have also been shown to disrupt social cognition in older adults and individuals with dementia (Schroeter et al., 2021).

Beyond face perception and emotion recognition, face masks may also influence face familiarity. The first study examining familiarity under masked conditions (Carragher and Hancock, 2020) found that face masks reduced performance in face-matching tasks, with similar impairments for personally familiar and unfamiliar faces. However, participants showed different error patterns, committing more false-positive errors for masked familiar faces and more false-negative errors for masked unfamiliar faces. In contrast, Kollenda and de Haas (2022) demonstrated that face familiarity confers a recognition advantage irrespective of face masks, using a face memory task. Similarly, Carlaw et al. (2022) found familiarity effects using a recognition-without-identification paradigm but reported no difference between occlusion caused by sunglasses and surgical masks.

Noyes et al. (2021) further examined the effects of masks and sunglasses on face matching and emotion recognition. While both occlusions impaired performance for unfamiliar faces, sunglasses did not impair matching for personally familiar faces, whereas masks did. The authors argued that masks occlude a larger facial area than sunglasses and suggested that differential experience with these forms of occlusion may contribute to the observed effects. This raises the possibility that prolonged exposure to masked faces may reduce mask-related impairments. However, evidence for such adaptation remains mixed. No improvement with exposure was reported for face matching (Freud et al., 2022) or emotion recognition (Carbon, 2020). In contrast, Carragher et al. (2022) demonstrated that diagnostic face training can improve masked face perception. Studies focusing on emotion perception have reported learning effects, with participants becoming more effective at using available visual cues over time (Barrick et al., 2021; Leitner et al., 2022). These findings challenge claims that face processing is entirely innate and resistant to environmental change (Carbon et al., 2022; Freud et al., 2022), suggesting instead that face masks may alter how visual information is utilized.

Neural studies using EEG have identified face-sensitive event-related potential (ERP) components associated with face perception. Early signatures include the P100 component (Dering et al., 2011; Debruille et al., 1998), although its role in face-specific processing remains debated (Bentin et al., 2010; Rossion and Jacques, 2008; Kuefner et al., 2010). The N170 component, a negative-going ERP peaking around 170 ms post-stimulus onset, is strongly associated with early structural encoding of faces and is typically larger for faces than non-face objects (Bentin et al., 2010; Joyce and Rossion, 2005). However, the sensitivity of the N170 to face familiarity is inconsistent across studies (Ramon and Gobbini, 2018). A recent review (Caharel and Rossion, 2021) proposed that for personally familiar faces, identity-specific processing may emerge between 150–200 ms in occipito-temporal regions.

Later ERP components show more consistent familiarity effects. The N250 component, occurring approximately 250 ms post-stimulus onset, has been robustly linked to face familiarity (Bentin and Deouell, 2000; Caharel et al., 2005; Paller et al., 2000; Pfütze et al., 2002; Tanaka et al., 2006; Pierce et al., 2011). N250 amplitudes are larger for personally familiar and self-faces than for unfamiliar faces, and scale with degree of familiarity (Herzmann et al., 2004; Huang et al., 2017).

Although numerous behavioral studies have examined masked face processing during COVID-19 (Carragher and Hancock, 2020; Carbon, 2020; Gori et al., 2021; Schroeter et al., 2021; Zochowska et al., 2022; Noyes et al., 2021; Prete et al., 2022), relatively few studies have investigated its neural correlates (Zochowska et al., 2022; Prete et al., 2022; Proverbio et al., 2023). Zochowska et al. (2022) reported enhanced P100 and P300 components and delayed P100 and N170 latencies for masked faces, with no N170 amplitude modulation, and similar effects across self, familiar, and unfamiliar faces. In contrast, Prete et al. (2022) found larger and delayed N170 responses for masked faces, as well as P200 modulation that diminished with greater mask exposure, suggesting neural adaptation. Using an emotion-word congruence paradigm, Proverbio et al. (2023) reported increased anterior P300 amplitudes for masked faces, reflecting higher mental load, and posterior P300 effects related to emotion categorization, with stronger effects for negative emotions.

Overall, face masks appear to hinder face processing and recognition, yet prolonged exposure to masked faces may confer perceptual benefits. We refer to this potential benefit as visual experience or visual exposure. The present exploratory study examined whether visual experience with masked faces influences face recognition across different levels of familiarity. Specifically, we investigated whether exposure to masked faces of personally familiar individuals improves masked face recognition. Personally familiar faces differ qualitatively from famous faces in that they involve long-term, real-life exposure to specific individuals, including repeated encounters with masked appearances.

Using both behavioral and EEG measures, we examined masked and unmasked face recognition for personally familiar, famous, and unfamiliar faces. While a longitudinal study reported no improvement in masked face recognition during the pandemic (Freud et al., 2022), a more recent study found faster and more accurate recognition of fully masked faces in the post-pandemic era (Chen and Wang, 2025). Crucially, the present study was conducted during the pandemic. Building on evidence that masked faces elicit increased N170 amplitudes reflecting greater processing effort (Prete et al., 2022), we aimed to clarify how exposure to masked faces, particularly of personally familiar individuals, modulates neural and behavioral responses.

Specifically, we addressed the following questions: (A) How do face masks affect face processing? (B) Does visual exposure to masked faces influence masked face perception? (C) Do the neural correlates of masked face perception differ from those of unmasked face perception, and how does face familiarity modulate these effects? We employed a 2-back face detection paradigm using Indian face stimuli comprising personally familiar, famous, and unfamiliar faces. Unlike simple detection tasks or paradigms presenting masked and unmasked faces simultaneously, the 2-back task introduces temporal separation and intervening identities, offering a more ecologically valid approximation of real-world face recognition.

## Materials and methods

###  Ethics statement

The study was carried out following institutional guidelines. All experimental protocols were approved by the Institute Ethics Committee of the Indian Institute of Science Education and Research (IISER), Kolkata, India. Participants gave written informed consent in accordance with the Declaration of Helsinki. We have also obtained informed consent for displaying representative images of the face stimuli used in our study.

###  Participants

41 healthy participants (age: 21–30 years, M: 24.45 years, SD: 2.68 years, 20 female and 21 male) participated in the behavioral study; of which we recorded EEG measurements from 25 participants (age (years): 22–30, M: 24.62, SD: 2.69, 12 female). We had initially planned to conduct only a behavioral experiment. However, on finding interesting trends in our behavioral data and acknowledging the gap in the literature concerning the neural underpinnings of masked-face perception in light of COVID-19, we decided to measure EEG responses for the same task. For this reason, the first 16 participants completed only the behavioral task, whereas for the remaining 25 participants, we also measured EEG data while they undertook the same behavioral task.

All participants had normal or corrected-to-normal vision, reported no history of neurological issues, and were naive to the purpose of the study. All participants were right-handed and used their right hands throughout the experiment to register their responses via mouse click.

The sample size for the behavioral task was estimated with the MorePower software (Campbell and Thompson, 2012) with estimation conducted for the main factors of mask (“unmasked face”, “masked face”) and familiarity (“personally familiar”, “famous”, “unfamiliar”) in a two-way repeated measures ANOVA model for estimated effect size partial η^2^ = 0.25, α = 0.05, β = 0.8. The computed results indicate a sample size of 24 participants. The EEG sample size (*N* = 25) was determined based on precedent in closely related ERP studies of face perception and masked-face recognition (Pierce et al., 2011; Prete et al., 2022; Thomas et al., 2025), which typically employ samples in the *N* = 12–20 range.

When our present study was first conceived during the second COVID wave in India (May 2021), face masks had been in abundant use for more than a year, and people seeing masked faces would plausibly have had the time to undergo adaptation, albeit possibly temporarily, toward the perception of masked faces. Residing in a secluded residential campus in the suburbs provided us with the unique opportunity to explore the effect of face masks and the associated adaptation effect on face familiarity. During the time of data collection in the current study, the campus was closed to the public and only a limited number of individuals resided on the campus who were personally familiar with each other and were continuously seeing other residents with face masks, especially at the dining area which was the only place food was available in the campus. Thus, the campus residents provided the ideal population to test the effect of possible adaptation to face masks. Participants were chosen such that they had spent at least 6 months on campus during the pandemic to ensure a high likelihood of them having seen the personally familiar faces in the masked state. We also used a post-experiment questionnaire where the participants rated their subjective familiarity level with each of the personally familiar stimuli shown to them. This is described in more detail in the following Procedure section.

###  Stimuli and display

The data set consisted of 2.67 inch x 4 inch (final viewing size) 8-bit gray-scale images of Indian famous, personally familiar, and unfamiliar faces in both unmasked and masked (face masks on) conditions. The participants were seated at a distance of approximately 30 inches from the display. Images were displayed at the center of the screen and subtended a visual angle of 5.10 degrees x 7.63 degrees. The display resolution of the display screen was set at 1366 x 768.

###  Stimuli preparation

Images of 20 individuals (10 females and 10 males) belonging to each familiarity category were used. Three different face images per individual, with variations in pose and gaze, were selected. Images of Indian famous faces licensed under the Creative Commons license were obtained from the internet. Images of unfamiliar faces were obtained from an Indian face database (Mayaluri et al., 2019) licensed under Creative Commons Attribution. Images of personally familiar faces were obtained through consensual digital capture of face photographs of research scholars and residents of IISER Kolkata. As described previously, the pool of personally familiar faces was selected from the population of students and staff residing on campus during the lockdown period. This ensured that the participants knew these individuals through regular interactions daily (such as, during meals at our only dining hall or in research building hallways) within an enclosed campus community at a time when traffic into and out of campus was restricted due to the pandemic. As further described in the Procedure, Analysis, and Results sections, participants also completed a post-experiment familiarity rating to indicate which faces from the “personally familiar” and “famous” categories they had known before participating in the study.

**Figure 1:**
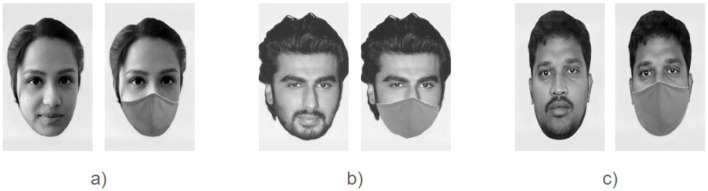
Sample images of faces used in the 2-back task, each shown in both unmasked and masked conditions: **(a)** personally familiar, **(b)** famous, and **(c)** unfamiliar face images (**a** - informed consent obtained for anonymous display of the images in the public domain; **b** - Bollywood Hungama, Arjun kapoor 12, CC BY 3.0, via Wikimedia Commons; **c** -images were made available in a public database: Mayaluri et al., 2019).

All the images were selected to ensure that they were devoid of accessories such as spectacles, sunglasses, hairbands, etc. To create the masked counterparts of each original image, a uniform cloth face mask was added to each image using a Python code (Anwar and Raychowdhury, 2020). The face masks in each image were manually adjusted and edited using Photoshop to make each face image appear as natural as possible. Any accessories still present in the images, such as earrings, were removed using Photoshop. After digitally adding face masks, all the images were refined using an image-editing software (Remini: AI Photo Enhancer, 2019) to match the image resolution across all images. All the images were then cropped to the same size at 283 x 425 pixels (72 pixels/inch) to keep only the face and the hair, keeping the face centered in the image. Next, the images were converted to grayscale with a uniform background. Finally, low-level image properties such as mean luminance and contrast were matched across all images using the SHINE toolbox (Willenbockel et al., 2010) run under MATLAB environment (ver. 2018b) (MATLAB, 2018). Specifically, the mean and standard deviation (contrast) of the luminance for each stimulus image were scaled to equate these low-level image properties across the set of stimulus images using the SHINE Toolbox. Sample images of the stimuli used in our experiment are shown in Figure 1. More example face stimuli images are shown in Supplementary Figures 1–3.

###  Design

The design consisted of two experimentally manipulated factors. The first factor was the mask condition (with two levels: unmasked and masked), and the second factor was the familiarity condition (with three levels: personally familiar, famous, and unfamiliar), thus forming six unique conditions: unmasked personally familiar, unmasked famous, unmasked unfamiliar, masked personally familiar, masked famous, and masked unfamiliar. The experimental task was a 2-back face detection task, wherein participants were required to register a "yes" response through a mouse click, as quickly and accurately as possible, whenever they detected the repetition of the same individual's face image in a 2-back manner. The participant's behavioral and EEG responses were recorded during the task. The behavioral performance in the task was analyzed using performance accuracy and reaction time measures, whereas the neural activity was analyzed using ERP components related to face perception and face processing.

###  Procedure

**Figure 2:**
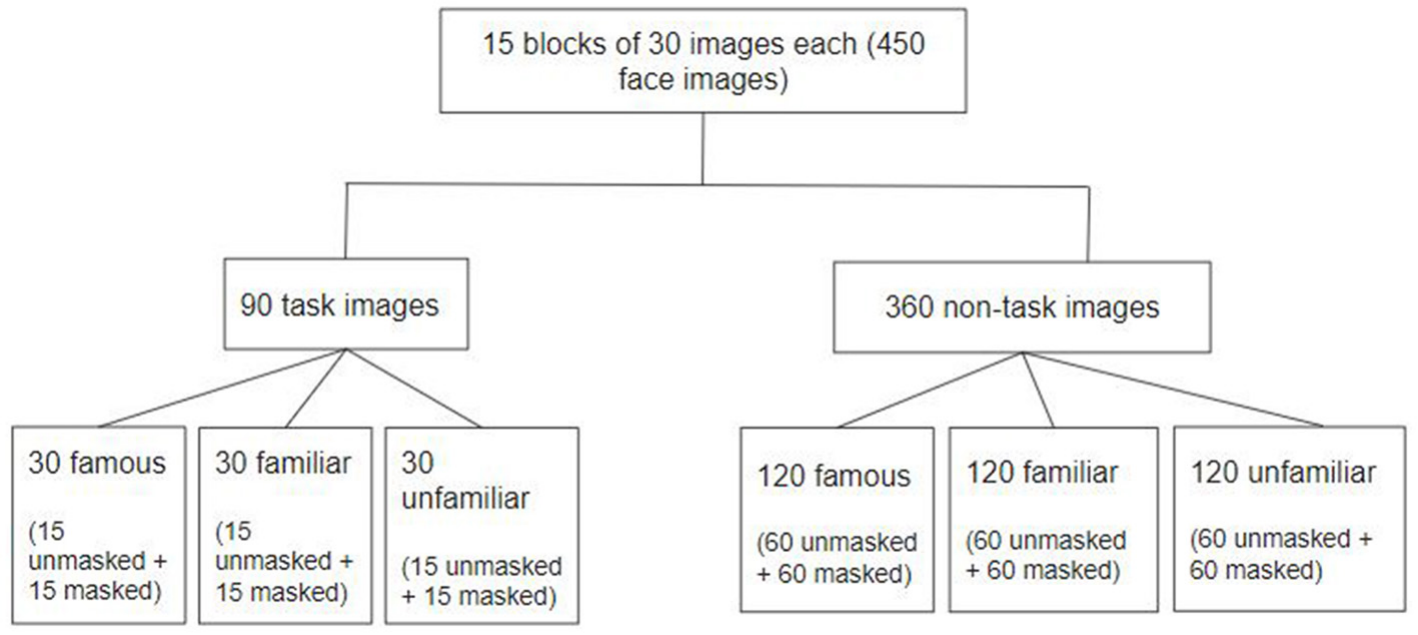
Experiment design: division of stimuli images of each familiarity and mask conditions into target and non-target trials.

**Figure 3:**
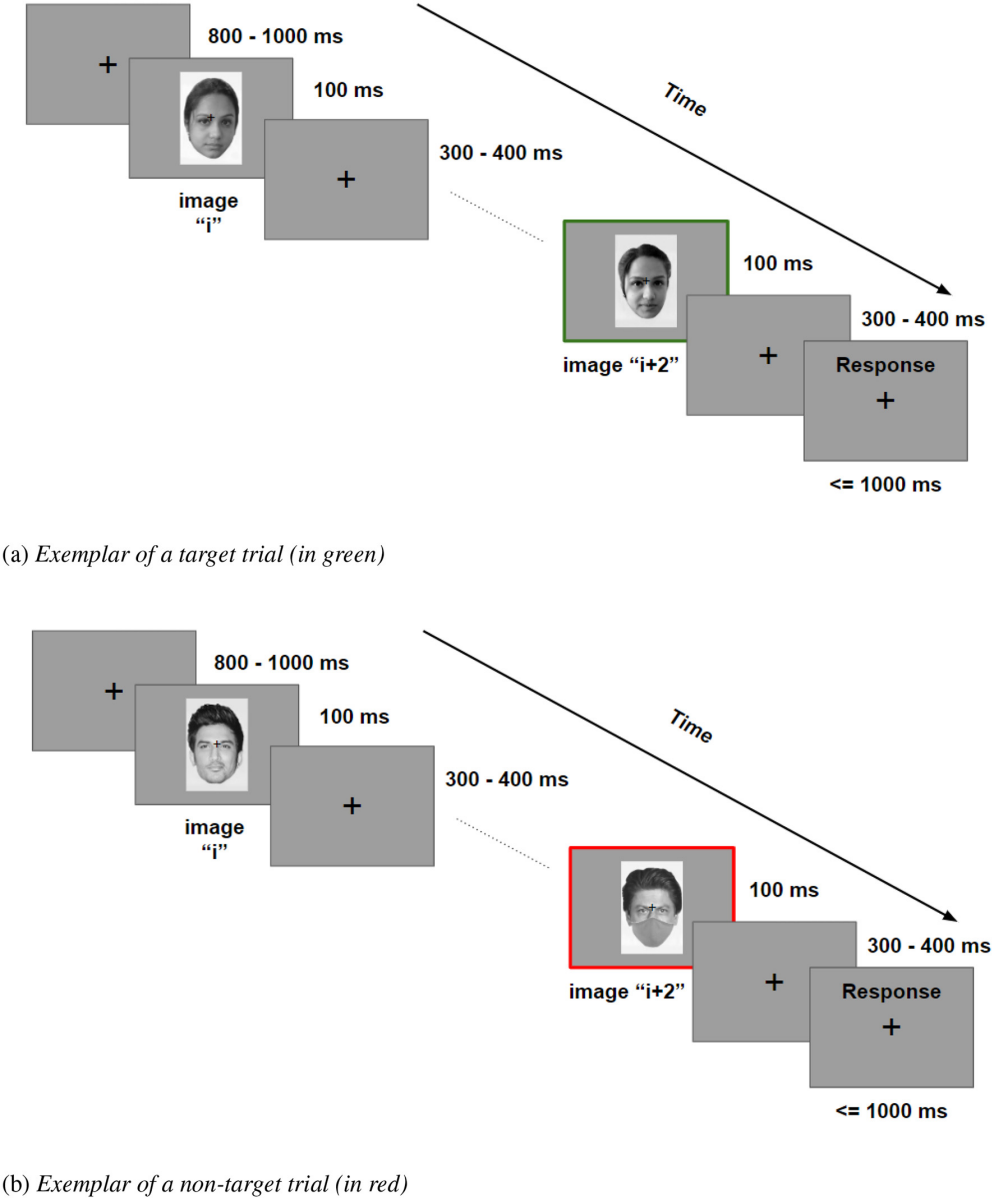
Experimental paradigm: 2-back task, showing **(a)** a target trial, and **(b)** a non-target trial (**a** - informed consent provided for anonymous display in the public domain; **b** Bollywood Hungama, Shushank Singh Rajput, CC BY 3.0, via Wikimedia Commons; Bollywood Hungama, Khan at audio release of JTHJ, CC BY 3.0, via Wikimedia Commons).

The experiment was based on a version of the n-back task paradigm (Owen et al., 2005), where *n* = 2. Within this 2-back task experimental design, participants were presented with a sequential display of 30 face images (trials) within a block and were required to respond with a left-click on the mouse whenever they believed that they found a repetition, in a 2-back manner, of an individual among the face images displayed. The experiment consisted of 450 trials split into 15 successive blocks performed in one sitting (see Figure 2). The total duration of the experiment was ~35 min. Each block had an equal representation of all six possible combinations of familiarity and mask conditions. At the beginning of the experiment, a practice block was run for the participants to adapt to the requirements of the task design. The practice block consisted of 30 trials, and a completely different set of stimulus images was used in the practice block. 20% of the trials were “target trial” pairs (see Figure 3a), wherein a true repetition of a stimuli-face occurred, and the rest 80% were “non-target trial” pairs (see Figure 3b), wherein the stimuli-face was not repeated in a two-back manner. Importantly, in target trials, the 2-back repeated faces were unique images of the same individual. In addition, for the target trials, the two different faces of the same individual forming the 2-back repeated pair were always either both unmasked or both masked. The sequence of trial presentation for each block was designed in a pseudo-random manner such that each of the six possible combinations of familiarity and mask conditions was uniformly represented in both target trials and non-target trials and across each of the 15 blocks (see Figure 2).

At the start of each block, instructions for the participants were listed on the screen for their convenience. Once ready, the participants clicked on this screen to start the experiment. Each trial started with a presentation of a fixation cross for 800-1000 ms (uniformly sampled between 800 and 1,000 ms), followed by the presentation of a face image for 100 ms, which was followed by a post-stimuli fixation cross for 300–400 ms (uniformly sampled between 300 and 400 ms). Next, for all trials except the first and the second of each block, a response screen was displayed with the text “Response” for a maximum of 1,000 ms, within which the participant was required to left-click on the mouse for target trials. There was only one response option: a left-click of the mouse, and participants were instructed to click only on target trials (2-back repeats). Upon a left-click of the mouse, the display screen moved on to the next trial. In case no left-click was registered, the response screen disappeared on the completion of 1000 ms, and the display screen automatically moved on to the next trial (Figure 3). The entire experimental paradigm was coded using PsychToolBox in the MATLAB environment (Brainard, 1997).

At the end of all 15 blocks, participants were required to complete a post-experiment familiarity rating task wherein participants were presented with gray-scale images of the personally familiar and famous individuals used as stimuli in the main experiment and were asked to indicate on a scale of 1–10 how familiar they were, at the time of participation, with the personally familiar and famous stimuli individuals. The images presented in the post-experiment familiarity rating task were the original unmasked and uncropped versions from which the respective stimulus image was generated, presented in grayscale. A rating of 10 indicated that the participant was certainly familiar with the personally familiar or famous individual, while a rating of 1 indicated that the participant was certainly unfamiliar with the given stimulus individual.

###  Behavioral performance measures

Owing to the experiment being a two-back task paradigm, four possible behavioral responses could be registered by a participant on each experimental trial: a true-positive (TP) response, wherein a participant clicks on a target trial; a true-negative (TN) response, in which a participant does not click on a non-target trial; a false positive (FP) response (or, a false alarm), wherein a participant clicks on a non-target trial; and a false negative (FN) response (or, a miss), in which a participant does not click on a target trial.

Two types of behavioral responses were collected as a measure of behavioral performance in the experimental task:

Performance Accuracy (PA): It is a measure of how well a participant performed in the above-mentioned experimental task, and is defined as


$$\begin{array}{l} PA=\frac{TP}{TP+FN} \end{array}$$ (1)

where, TP and FN are the total number of true positive and false negative responses, respectively, registered by the participant over all 15 experimental blocks.

Reaction Time (RT): It is calculated as the time between the onset of an image and the moment when the participant registers a click. This was measured using functions available in Psychtoolbox, MATLAB (Brainard, 1997). Although RTs were measured every time a participant registered a left-click on the mouse (that is, for TP as well FP responses), only those RTs corresponding to the TP responses were analyzed for this study because RT as a behavioral measure was intended to serve as a measure of how well a participant performs in the above-mentioned experimental task. In our experimental design, participants were required to register a “yes” response if they felt that they detected the repetition of the same individual in a 2-back manner by clicking the left mouse button as quickly as possible. Specifically, there was a 1-second time limit within which a response had to be registered before the screen moved on to the next trial automatically. This was done to ensure a quick response instead of a deliberative one. Having a time limit on the response screen also ensured that we did not have any outliers for the measure of reaction time in our data. Thus, we analyzed the collected reaction time in terms of the average RT per condition.

Finally, we also report signal decision theory measures of sensitivity (*d*′), or the standardized difference between the means of the response distributions of signal present and signal absent trials; and criterion (λ), or the level of evidence needed for an observer to make a signal present decision (see Wickens, 2002).

###  Neural data acquisition and preprocessing

EEG activity was measured using a 64-channel active shielded electrode setup (Nexus 64 system, Netherlands) mounted on an EEG cap which follows the International 10–20 system. EEG data were collected at a sampling rate of 512 Hz. Trials were band-pass filtered, to filter out those neural signals with frequencies lower than 0.1 Hz or greater than 40 Hz. The EEG signals, being a continuous measure of brain activity, were epoched with respect to stimulus onset and were referenced using average referencing. These preprocessed EEG epochs were time-locked to stimulus onset and consisted of a 200 ms pre-stimulus baseline and a 400 ms post-stimulus interval, and are referred to as EEG trials. Trials affected by eye movements, blinking, or motor movements were rejected using a semi-automatic approach with initial epoch rejection using EEGLAB toolbox (Delorme and Makeig, 2004) [run under MATLAB (MATLAB, 2018) environment], followed by manual rejection of noisy trials.

###  Data analysis

The experiment intended to test the effect of two experimental factors, namely, familiarity and face mask, on the performance of human participants in a 2-back task. In this context, the experimental conditions were decided as follows: For the familiarity factor, the three experimental conditions were famous, personally familiar, and unfamiliar (control). For the mask factor, the two experimental conditions were masked (with a face mask), and unmasked (without a face mask, control). The behavioral data were collected in the form of performance accuracy and reaction time, whereas the neural data were in the form of EEG signals, which were analyzed in the time domain. Thus, the dependent variables were performance accuracy and reaction time, for the behavioral data, and EEG potentials for the neural data. The independent variables, in both cases, were familiarity and mask conditions. All the analyses were run in MATLAB.

#### Behavioral data analysis

The performance accuracy for each participant was calculated as shown in Equation 1. In such an experimental paradigm, wherein each target trial has only two possible outcomes (a mouse-click, or no mouse-click), a chance-level performance accuracy (that is, if the participant were to randomly click on the mouse button for each trial) would be 50%. Participants with chance-level or lower performance accuracy were not considered for further analysis. Thus, 5 out of 41 participants, who had an average performance accuracy lower than 50% across all trials of unmasked personally familiar and unmasked famous faces, were removed from further analysis. For the remaining 36 participants, the post-experiment familiarity rating was used to determine which trials were fit for further analysis. In other words, all those trials that consisted of personally familiar and famous individuals that the participant rated lower than 6 in the post-experiment familiarity questionnaire were not analyzed further for either behavioral or neural responses. For the famous individuals, 7 of the 36 participants rated 5–15% of the famous individuals lower than 6 on the subjective familiarity rating, and the rest rated all the famous individuals greater than or equal to 6. In the case of the personally familiar individuals, 4 of the 36 participants rated all the personally familiar individuals greater than 6, and only 2 participants rated less than 25% of the personally familiar individuals greater than 6. We accounted for the subjective familiarity of each participant to the stimuli individuals shown and only analyzed those trials in the famous and personally familiar conditions that consisted of individuals with whom the participant reported a high subjective familiarity. This resulted in 164–191 (M = 181.33, SD = 13.15) true-positive (hit) trials per condition across all participants that contributed to the computation of performance accuracy and RT measures. Next, a two-way repeated measures ANOVA (Schurger, 2026) analysis was run to check for the effects of the two factors of familiarity (with 3 levels) and mask-condition (with 2 levels) on each of the two behavioral measures: performance accuracy and RT as well as on the proportion of false-positive responses. In case a significant main effect was observed in the ANOVA, post-hoc analyses were done using Tukey's HSD test to check for multiple comparisons.

#### Neural data analysis

Five of the twenty-five participants whose neural data were recorded had noisy EEG measurements, and two had below 50% (chance) accuracy. Thus, we did not analyze the EEG data from 7 of the 25 participants, and our final sample size for the EEG analysis was 18 participants. To clarify the statistical sensitivity of our final EEG sample size of 18 participants, we conducted a sensitivity analysis in G*Power (Faul et al., 2007) (F tests: two-way repeated-measures ANOVA, within factors). Assuming α = 0.05 and a final analyzed sample of *N* = 18 participants, the design had 80% power to detect a Cohen's effect size (f) = 0.25, corresponding to a partial η^2^ = 0.059. Thus, the EEG sample was sufficiently powered to detect medium-sized within-subject effects in the present design.

After performing epoch rejection on the 164–191 (M = 181.33, SD = 13.15) true-positive (hit) trials per condition across all participants that contributed to our behavioral analyses, we had 148–181 (M = 167.33, SD = 11.33) trials per condition over all participants that contributed to our neural ERP analyses. Neural analysis focused on ERP components of P100, N170, and N250. For the N170 and N250 ERP components, the left-brain and right-brain electrode clusters were chosen as TP7, P7, P9, PO3, and PO7, and TP8, P8, P10, PO4, and PO8, respectively (Tanaka et al., 2006; Pierce et al., 2011; Zochowska et al., 2022). Whereas, for the P100 ERP components, the electrode clusters were chosen as O1, O2, PO3 and PO4, respectively (Zochowska et al., 2022).

For each trial, voltages were averaged across electrodes within the predefined cluster as described above. Condition-wise ERPs were then obtained by averaging these cluster-averaged waveforms across trials. Finally, for statistical analyses, we extracted peak-based amplitude from the resulting cluster averages using a local maxima detector in MATLAB (findpeaks) within the 80–140 ms time window for the P100 and the 110–200 ms time window for the N170 components. For the N250 component, we computed the mean amplitude within the 230-320 ms time window (Tanaka et al., 2006; Pierce et al., 2011). As described in previous literature (Pierce et al., 2011), a mean amplitude approach was used for the N250 component rather than detecting peaks as for N170 because N250 peaks are difficult to measure reliably in individual subjects.

For each participant, all (task as well as non-task) EEG trials were segregated into the 6 conditions (combination of the factors of familiarity and masking), and for each condition, the EEG trials were averaged to give participant-wise ERPs. The mean of each participant-averaged ERP was calculated to generate the grand-averaged ERPs for each condition. Those target trials of the 2-back task paradigm on which the participant had performed correctly (true positive trials, interchangeably referred to as correct target trials) were analyzed in terms of their corresponding ERP amplitudes. The computed ERP components were tested for significance using one-way and two-way repeated-measure ANOVA models. Using a two-way ANOVA with mask and familiarity as the two factors for ERP analysis, however, resulted in a significant interaction effect (Meyers et al., 2016) for the N170 and N250 amplitudes, thereby rendering the statistical inference of the main effect uninterpretable. Thus, we followed this up with one-way repeated measures ANOVA tests, equivalent to performing post-hoc Tukey's tests (see Christensen, 2018, page 340). We used five one-way repeated measures ANOVA by fixing each of the five levels (Unmasked, Masked, Personally Familiar, Famous, and Unfamiliar) of the two independent variables (Mask and Familiarity). Finally, if a significant main effect was present in the one-way ANOVA models, follow-up analyses using two-way repeated measures ANOVA with hemisphere (left and right) as an additional factor were run.

#### Multivariate pattern analysis

Multivariate pattern analysis (MVPA) of EEG data considers the relationships and dependencies between multiple channels (e.g., EEG electrodes) simultaneously, allowing it to infer spatial patterns and interactions that univariate methods fail to capture. To leverage the spatiotemporal richness of EEG signals, we therefore employed MVPA. In this study, MVPA was performed following a channel selection technique based on Functional Data Analysis (FDA) (Das et al., 2023). While ERP components were examined using clusters of electrodes previously identified in the literature as relevant for face recognition, the MVPA electrode selection relied solely on the output of the FDA-based channel selection algorithm, without incorporating any prior domain knowledge. The channel selection method used operates in two stages. First, it identifies electrodes with high neural activity. Then, in the second stage, these active electrodes are ranked based on the discriminative information they provide for distinguishing between classes. Using these rankings and the functional correlations between electrodes, the algorithm forms ranked clusters. From each cluster, a fixed number of electrodes is selected to preserve essential information while minimizing redundancy from correlated signals. We have classified between masked and unmasked conditions for all three familiarity categories (personally familiar, famous, and unfamiliar). After the channel selection, we used a linear support vector machine (SVM) classifier to classify the single-trial EEG signals. A 10-fold cross-validation method was used for each participant to classify the trials, and then the mean area under the curve (AUC) across participants is reported in Supplementary Figure 4. Statistical significance was tested using parametric testing along with Bayes Factor analysis.

###  Transparency and openness statement

The present study was not preregistered. We report the processes we used to determine our sample size, any data exclusions, all experimental manipulations, and all measures. The data for this study was collected between October 2021 and March 2022. The sample of subjects consisted of Indian university undergraduates and graduate students between 21 and 30 years old. The raw and processed behavioral data, processed EEG data, and codes for experiment presentation, analysis, and figure generation are made publicly available on a GitHub repository. The large raw EEG dataset (~15 GB) is hosted on Zenodo repository.

## Results

###  Behavioral results

Two-way repeated measures ANOVA was used to test the effects of the two manipulated factors of familiarity and mask on behavioral performance in terms of performance accuracy, RT, and the proportion of false-positive responses. ANOVA tests were required to be followed by multiple comparison tests because there were more than two levels of the familiarity factor. A *post-hoc* Tukey's test was chosen as the multiple comparison test to identify which of the means among the experimental conditions are significantly different from each other. The results for each of the three dependent variables are described in the following sections.

#### Performance accuracy

The two-way repeated measures ANOVA revealed significant main effects of familiarity [F_(2, 70)_ = 10.07, *p* < 0.001, partial η^2^ = 0.223, BF = 2.63] as well as mask [F_(1, 35)_ = 12.44, *p* = 0.001, partial η^2^ = 0.262, BF = 39.74] on performance accuracy. The interaction effect was non-significant [F_(2, 70)_ = 2.12, *p* = 0.127, partial η^2^ = 0.057, BF = 0.08] (see Supplementary Tables 1, 3). The significant main effect of mask was observed in the greater mean performance accuracy for unmasked faces (M = 73.86%, SE = 1.1%) than for masked faces (M = 67.47%, SE = 0.2%), collapsing across the levels of the familiarity factor.

**Figure 4:**
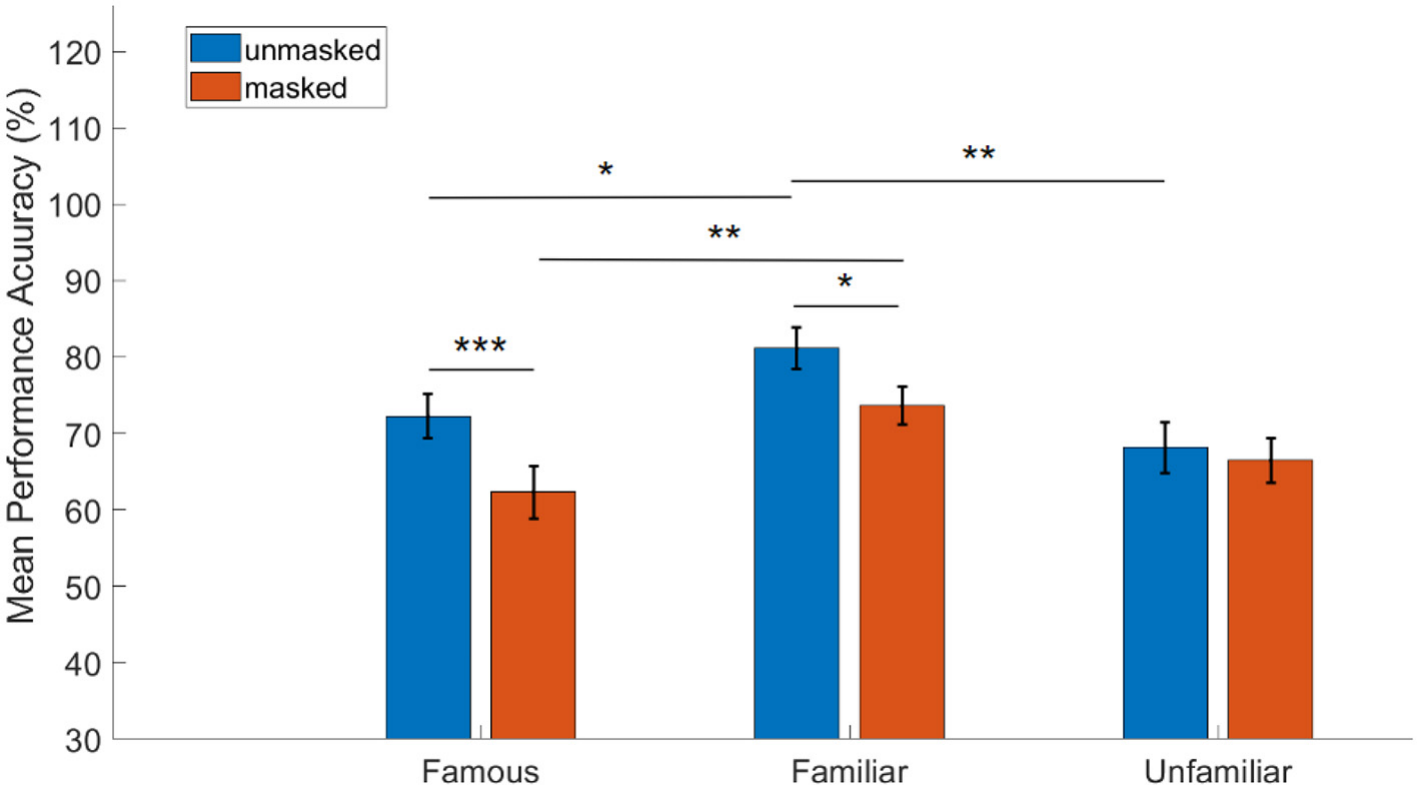
Effect of familiarity and face mask on performance accuracy: results of two-way repeated measures ANOVA followed by Tukey's multiple comparison tests (*N* = 36, error bars indicate standard error of the mean ***p<0.001, **p<0.01, *p<*0.05*).

The ANOVA was followed by a *post-hoc* Tukey's test to further investigate the significant main effect of familiarity (see Supplementary Table 2). The results are described as follows (see Figure 4): for famous faces, the mean performance accuracy for unmasked condition (M = 72.27%, SE = 2.9%) was significantly higher than that for the masked condition (M = 62.33%, SE = 3.5%) (*p* < 0.001, 95% CI = [0.04, 0.15], BF = 39.85). For personally familiar faces, although mean performance accuracy for unmasked condition (M = 81.17%, SE = 2.72%) was significantly greater than that for masked condition (M = 73.61%, SE = 2.47%) (*p* = 0.02, 95% CI = [0.01, 0.14], BF = 2.14), the Bayes Factor (BF) was comparatively lower than that obtained for famous faces. Finally, for unfamiliar faces, the difference in performance accuracy between unmasked (M = 68.15%, SE = 3.3%) and masked (M = 66.48%, SE = 2.89%) conditions was not statistically significant.

Further, mean performance accuracy was found to be significantly lower for unmasked famous faces (M = 72.27%, SE = 2.9%) than for unmasked personally familiar faces (M = 81.17%, SE = 2.72%) (*p* = 0.02, 95% CI = [-0.16,-0.01], BF = 6.02). This reduction was larger between masked famous faces (M = 62.33%, SE = 3.5%) and masked personally familiar faces (M = 73.61%, SE = 2.47%) (*p* = 0.003, 95% CI = [-0.19,-0.04], BF=30.92). Mean performance accuracy was also found to be significantly higher for unmasked personally familiar faces (M = 81.17%, SE = 2.72%) than for unmasked unfamiliar faces (M = 68.15%, SE = 3.3%) (*p* = 0.004, 95% CI = [0.04,0.22], BF = 23.6). No other pairwise comparison was statistically significant (see Supplementary Table 2 for details).

#### Reaction time (RT)

The two-way repeated measures ANOVA revealed significant main effects of familiarity [F_(2, 70)_ = 12.62, *p* < 0.001, partial η^2^ = 0.265, BF = 7.09] as well as mask [F_(1, 35)_ = 10.73, *p* = 0.002, partial η^2^ = 0.235, BF = 20.52] on RT. The interaction effect was non-significant [F_(2, 70)_ = 2.16, *p* = 0.123, partial η^2^ = 0.058, BF = 0.08] (see Supplementary Tables 4, 6). The significant main effect of mask was observed in the lower mean RT for unmasked faces (M = 0.26 s, SE = 0.01 s) than for masked faces (M = 0.3 s, SE = 0.01 s), collapsing across the levels of the familiarity factor.

**Figure 5:**
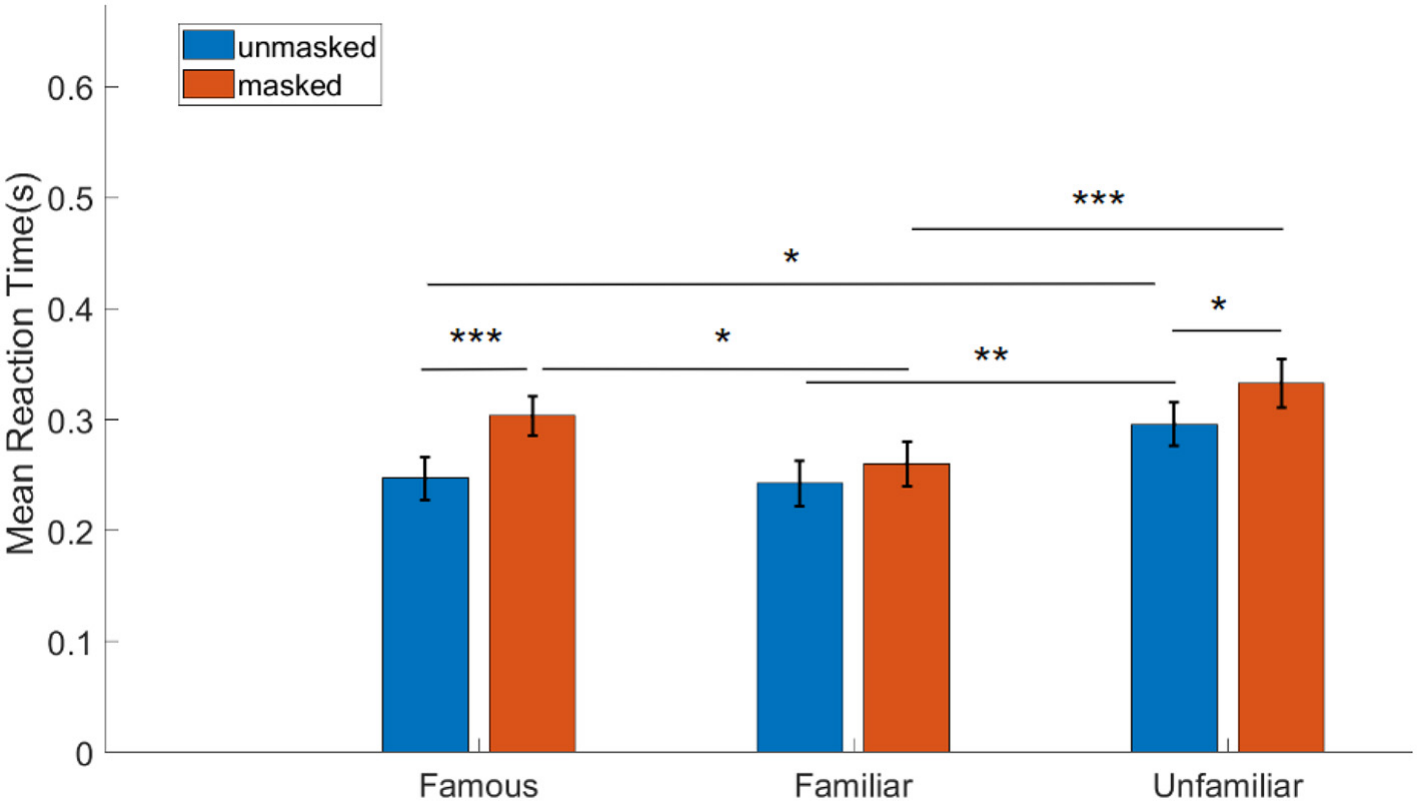
Effect of familiarity and face mask on reaction time: results of two-way repeated measures ANOVA followed by Tukey's multiple comparison test (*N* = 36, error bars indicate standard error of mean, ***p<0.001, **p<0.01, *p<*0.05*).

The ANOVA was followed by a post-hoc Tukey's test to further investigate the significant main effect of familiarity (see Supplementary Table 5). The results are described as follows (see Figure 5): for famous faces, mean RT for unmasked condition (M = 0.25 s, SE = 0.02 s) was significantly lower than that for masked condition (M = 0.3 s, SE = 0.02 s) (*p* < 0.001, 95% CI = [-0.09,-0.03], BF = 43.42). For personally familiar faces, mean RT between unmasked (M = 0.24 s, SE = 0.02 s) and masked (M = 0.26 s, SE = 0.02 s) conditions was not significantly different. Finally, for unfamiliar faces, mean RT for unmasked condition (M = 0.29 s, SE = 0.02 s) was significantly lower than that for masked condition (M = 0.33 s, SE = 0.02 s) (*p* = 0.01, 95% CI = [-0.07,-0.01], BF = 3.61).

Further, mean RT was also found to be significantly lower for unmasked personally familiar (M = 0.24 s, SE = 0.02 s) than for unmasked unfamiliar (M = 0.29 s, SE = 0.02 s) (*p* = 0.003, 95% CI = [-0.09,-0.02], BF = 30.66). Mean RT was also significantly lower for unmasked famous faces (M = 0.25 s, SE = 0.02 s) than for unmasked unfamiliar faces (M = 0.29 s, SE = 0.02 s) (*p* = 0.01, 95% CI = [-0.09,-0.01], BF = 8.06). Mean RT was found to be significantly higher for masked famous faces (M = 0.3 s, SE = 0.02 s) than for masked personally familiar faces (M = 0.26 s, SE = 0.02 s) (*p* = 0.04, 95% CI = [0.002,0.08], BF = 3.13). There was a strong significant increase in mean RT for masked unfamiliar faces (M = 0.33 s, SE = 0.02 s) when compared to masked personally familiar faces (M = 0.26 s, SE = 0.02 s) (*p* < 0.001, 95% CI = [-0.11,-0.04], BF = 991.83). No other pairwise comparison was statistically significant (see Supplementary Table 5 for details).

#### False positive (FP) response

The two-way ANOVA revealed significant main effects of familiarity [F_(2, 70)_ = 93.77, *p* < 0.001, partial η^2^ = 0.728, BF = 4.24 × 10^8^] as well as mask-condition [F_(1, 35)_ = 32.98, *p* < 0.001, partial η^2^ = 0.485, BF = 2.58 × 10^4^] on the proportion of FP responses. The interaction effect was non-significant [F_(2, 70)_ = 0.41, *p* = 0.662, partial η^2^ = 0.011, BF = 0.04] (see Supplementary Tables 7, 9). The significant main effect of mask was observed in the lower mean proportion of false-positive responses for unmasked faces (M = 0.02, SE = 0.005) than for masked faces (M = 0.04, SE = 0.005), collapsing across the levels of the familiarity factor.

**Figure 6:**
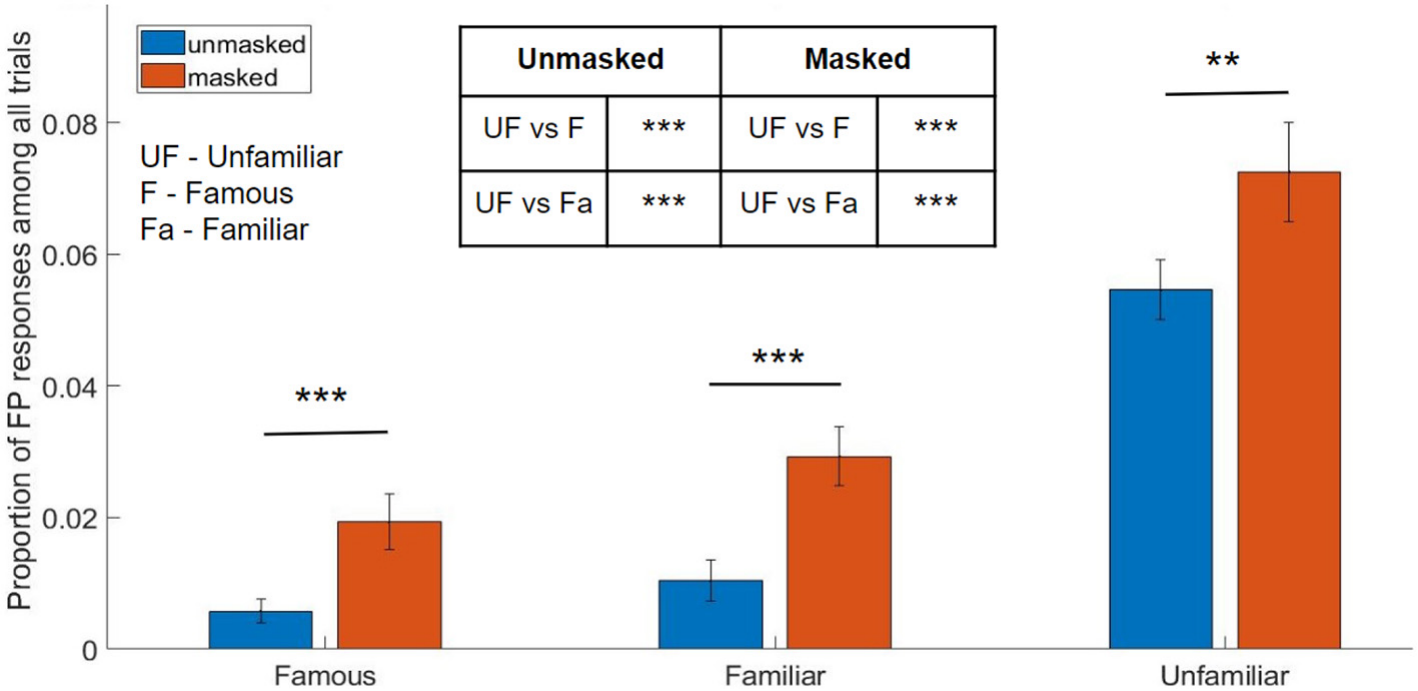
Proportion of false positive responses (error bars indicate standard error of the mean, **p<0.01, ***p<*0.001*).

The ANOVA was followed by a post-hoc Tukey's test to further investigate the significant main effect of familiarity (see Supplementary Table 8). The results are described as follows (see Figure 6): For famous faces, the proportion of FP responses for unmasked condition (M = 0.006, SE = 0.002) was significantly lower than that for masked condition (M = 0.019, SE = 0.004) (*p* < 0.001, 95% CI = [-0.02,-0.01], BF = 133.68). For personally familiar faces, again, the proportion of FP responses was significantly lower in the unmasked condition (M = 0.01, SE = 0.003) than in the masked condition (M = 0.029, SE = 0.004) (*p* < 0.001, 95% CI = [-0.03,-0.01], BF = 212.15). Finally, for unfamiliar faces, the proportion of FP responses was again significantly lower in the unmasked condition (M = 0.055, SE = 0.004) than in the masked condition (M = 0.073, SE = 0.007) (*p* = 0.006, 95% CI = [-0.03,-0.01], BF = 6.25).

Further, the proportion of FP responses was also found to be significantly lower for unmasked personally familiar (M = 0.01, SE = 0.003) than for unmasked unfamiliar (M = 0.055, SE = 0.004) faces (*p* < 0.001, 95% CI = [-0.05,-0.03], BF = 2.67^10^). FP responses were also lower for unmasked famous faces (M = 0.006, SE = 0.002) than for unmasked unfamiliar faces (M = 0.055, SE = 0.004) (*p* < 0.001, 95% CI = [-0.06,-0.04], BF = 1.52^10^). FP response proportion was found to be significantly lower for masked famous faces (M = 0.019, SE = 0.004) than for masked unfamiliar faces (M = 0.073, SE = 0.007) (*p* < 0.001, 95% CI = [-0.07,-0.04], BF = 8.05^6^). FP response proportion was also found to be significantly lower for masked personally familiar faces (M = 0.029, SE = 0.004) as compared to masked unfamiliar faces (M = 0.073, SE = 0.007) (*p* < 0.001, 95% CI = [-0.06,-0.03], BF = 1.48^4^) (see Supplementary Table 8 for details).

#### Signal detection theory measures: sensitivity

The two-way repeated measures ANOVA revealed significant main effects of familiarity [F_(2, 70)_ = 44.78, *p* < 0.001, partial η^2^ = 0.561, BF = 7.67 × 10^4^], mask [F_(1, 35)_ = 37.98, *p* = 0.001, partial η^2^ = 0.52, BF = 9.25 × 10^4^], and a significant interaction effect on sensitivity [F_(2, 70)_ = 4.94, *p* = 0.01, partial η^2^ = 0.124, BF = 0.3] (see Supplementary Table 10).

**Figure 7:**
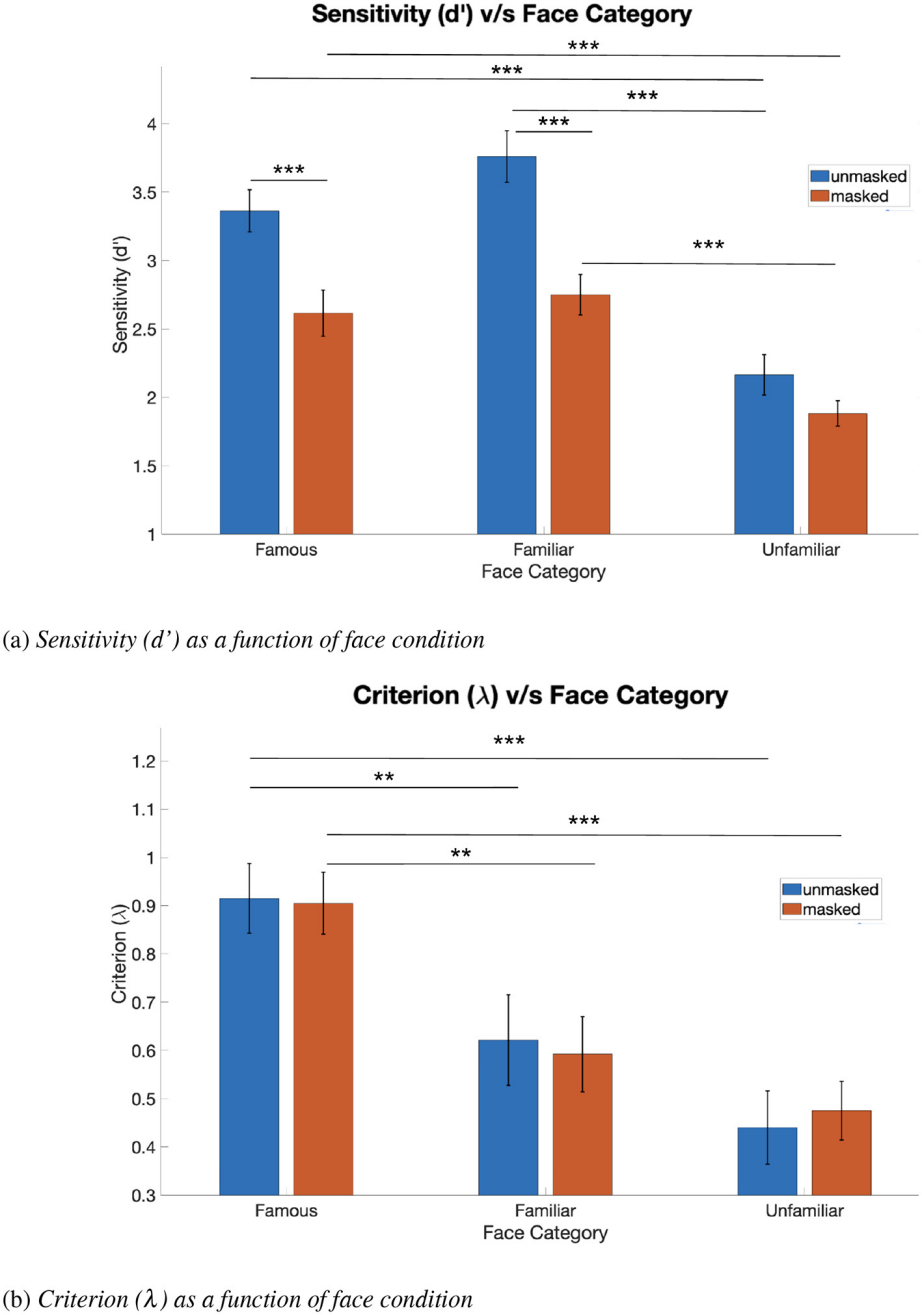
Signal detection theory measures: **(a)** sensitivity and **(b)** criterion: results of two-way repeated measures ANOVA followed by Tukey's multiple comparison tests (*N* = 36, error bars indicate standard error of the mean, **p < 0.01, ***p < 0.001).

The ANOVA was followed by a *post-hoc* Tukey's test to further investigate the significant main interaction effect. The results are described as follows (see Figure 7a): for famous faces, the mean sensitivity for unmasked condition (M = 3.363, SE = 0.154) was significantly higher than that for the masked condition (M = 2.615, SE = 0.169) (*p* < 0.001, 95% CI = [0.292, 1.204], BF = 552.15). For personally familiar faces as well, the mean sensitivity for the unmasked condition (M = 3.761, SE = 0.189) was significantly greater than that for the masked condition (M = 2.750, SE = 0.147) (*p* < 0.001, 95% CI = [0.53,1.49], BF = 718.59). Finally, for unfamiliar faces, the difference in mean sensitivity between unmasked (M = 2.164, SE = 0.147) and masked (M = 1.882, SE = 0.093) conditions was not statistically significant.

Further, mean sensitivity was found to be significantly higher for unmasked personally familiar faces (M = 3.761, SE = 0.189) than for unmasked unfamiliar faces (M = 2.164, SE = 0.147) (*p* < 0.001, 95% CI = [1.12, 2.07], BF = 9.37^5^). Mean sensitivity was significantly higher for unmasked famous faces (M = 3.363, SE = 0.154) than for unmasked unfamiliar faces (M = 2.164, SE = 0.147) (*p* < 0.001, 95% CI = [0.77, 1.62], BF = 3.84^5^).

Finally, mean sensitivity was found to be significantly higher for masked famous faces (M = 2.615, SE = 0.169) than for masked unfamiliar faces (M = 1.882, SE = 0.093) (*p* < 0.001, 95% CI =[0.35, 1.12], BF = 318.95). Mean sensitivity was also found to be significantly higher for masked personally familiar faces (M = 2.750 SE = 0.147) than for masked unfamiliar faces (M = 1.882, SE = 0.093) (*p* < 0.001, 95% CI = [0.52, 1.21], BF = 2.66^4^). No other pairwise comparisons were significantly different from each other.

The trends in the results of sensitivity closely reflect those in the measure of performance accuracy.

#### Signal detection theory measures: criterion

The two-way repeated measures ANOVA revealed a significant main effect of familiarity [F_(2, 70)_ = 24.12, *p* < 0.001, partial η^2^ = 0.41, BF = 3.48 × 10^2^]. The main effect of mask and the interaction effect were both non-significant (see Supplementary Table 11).

The ANOVA was followed by a post-hoc Tukey's test. The results are described as follows (see Figure 7b): the mean criterion was found to be significantly higher for unmasked famous faces (M = 0.915, SE = 0.072) than for unmasked personally familiar faces (M = 0.621, SE = 0.094) (*p* < 0.01, 95% CI = [0.058, 0.53], BF=50.49). Mean criterion was also significantly higher for unmasked famous faces (M = 0.915, SE = 0.072) than for unmasked unfamiliar faces (M = 0.440, SE = 0.076) (*p* < 0.001, 95% CI = [0.27, 0.68], BF =7.78^4^).

Further, the mean criterion was significantly larger for masked famous faces (M = 0.905, SE = 0.064) than for masked personally familiar faces (M = 0.592, SE = 0.078) (*p* < 0.01, 95% CI = [0.11, 0.51], BF = 70.66). The mean criterion was also significantly larger for masked famous faces (M = 0.905, SE = 0.064) than for masked unfamiliar faces (M = 0.475, SE = 0.061) (*p* < 0.001, 95% CI = [0.25, 0.61], BF = 6.52^5^). No other pairwise comparisons were significantly different from each other.

These results show that participants were more conservative in their judgments of famous faces as compared to personally familiar and unfamiliar faces, across masked and unmasked conditions. Importantly, inclusion of face masks did not result in a criterion shift for any of the three face familiarity categories.

#### Behavioral results summary

There was an overall significant effect of the factor of face mask as well as the factor of familiarity. Personally familiar face recognition elicited the best performance, followed by famous face recognition, irrespective of the face mask category. Additionally, reaction times were fastest for personally familiar faces than for famous faces and unfamiliar faces. The masked condition resulted in significantly lower performance accuracy and higher reaction time for famous face recognition when compared with its unmasked counterpart. There was no significant change in terms of performance in the unfamiliar face identity for masked and unmasked conditions; however, reaction time for unmasked unfamiliar faces was significantly lower than its masked counterpart. In the context of personally familiar face recognition, the masked condition showed reduced performance accuracy, and there was no significant difference in reaction time between masked and unmasked personally familiar face recognition. Masked unfamiliar face condition resulted in the highest false positive errors, pointing to the inherent difficulty in processing unknown faces compared to personally familiar faces. These results are indicative of the possible role of visual experience in identifying frequently viewed masked faces.

Behavioral measures of performance accuracy and sensitivity aligned in their trends; such that participants showed higher sensitivity (and accuracy) for unmasked personally familiar and famous faces as compared to their masked counterparts, as well as a higher sensitivity (and accuracy) for personally familiar and famous faces as compared to unfamiliar faces, across unmasked and masked conditions. Importantly, evidence from reaction time data supported our hypothesis that personally familiar faces, which one encounters in the masked condition in one's day-to-day life, are faster recognized in the masked condition than other categories of known faces, such as faces of celebrities. Further, even with similar sensitivity to personally familiar and famous faces, and with faster reaction times for personally familiar faces, participants were more accurate in personally familiar face recognition than in famous face recognition, regardless of unmasked and masked conditions. In other words, participants were significantly slowed by masked famous faces and were significantly less accurate, as compared to unmasked famous faces. Critically, this points toward an advantage for masked personally familiar face recognition in contrast to masked famous face recognition, owing to a higher exposure to the former category of faces.

###  ERP results

The event-related potentials of the three early components (P100, N170, and N250) was analyzed for true positive responses.

#### P100

**Figure 8:**
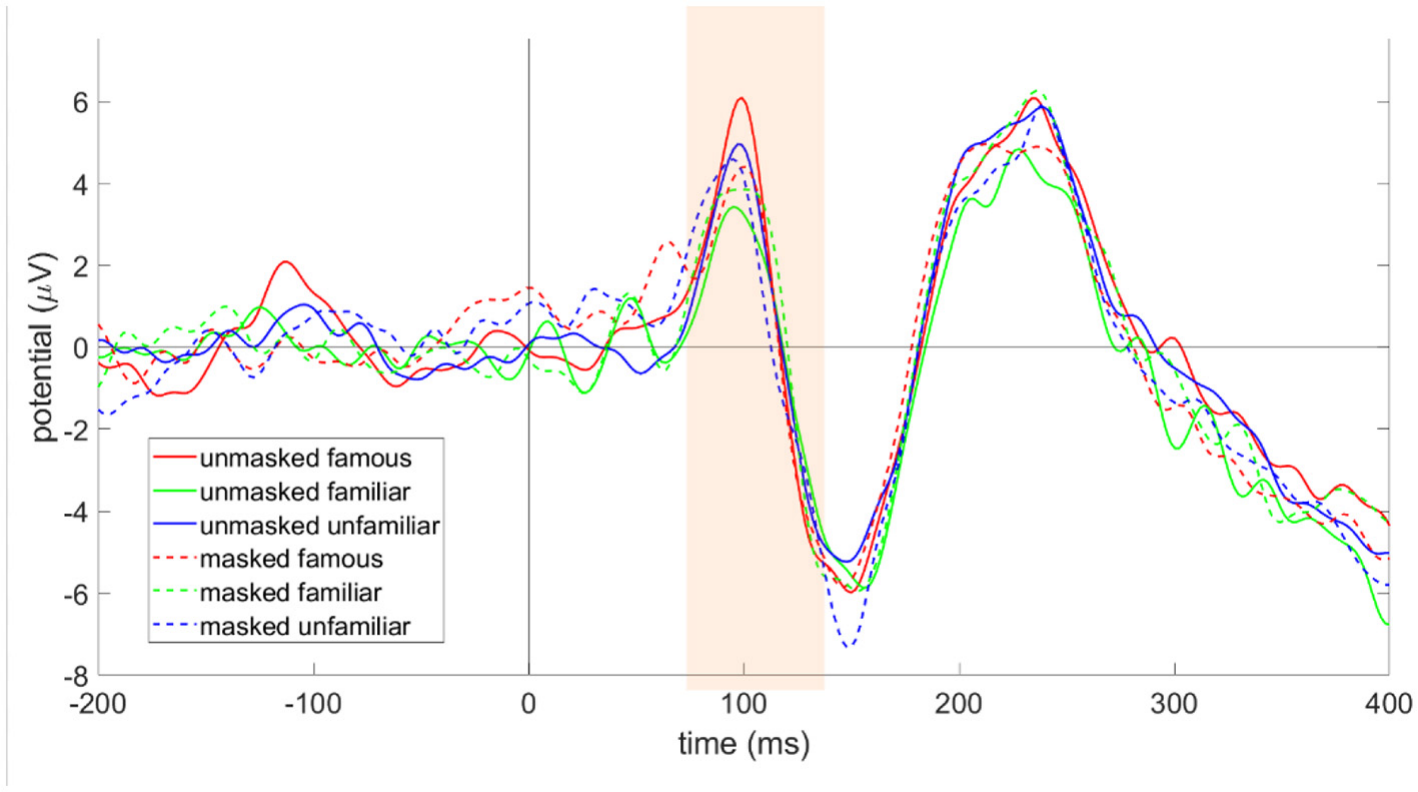
P100: Grand-averaged ERP across all electrodes in the cluster (O1, O2, PO3, PO4) (*N* = 18).

The P100 component displayed no significant main effect of familiarity [F_(2, 34)_ = 1.34, *p* = 0.276, partial η^2^ = 0.005, BF = 0.11] and mask [F_(1, 17)_ = 0.08, *p* = 0.777, partial η^2^ = 0.077, BF = 0.25] condition with insignificant interaction effect ([F_(2, 34)_ = 2.57, *p* = 0.092, partial η^2^ = 0.139, BF = 0.2], in a two-way repeated measure ANOVA model (Figure 8). The result is also consistent when looked at separately for the left and right hemisphere clusters (see Supplementary Tables 12–14).

#### N170

**Figure 9:**
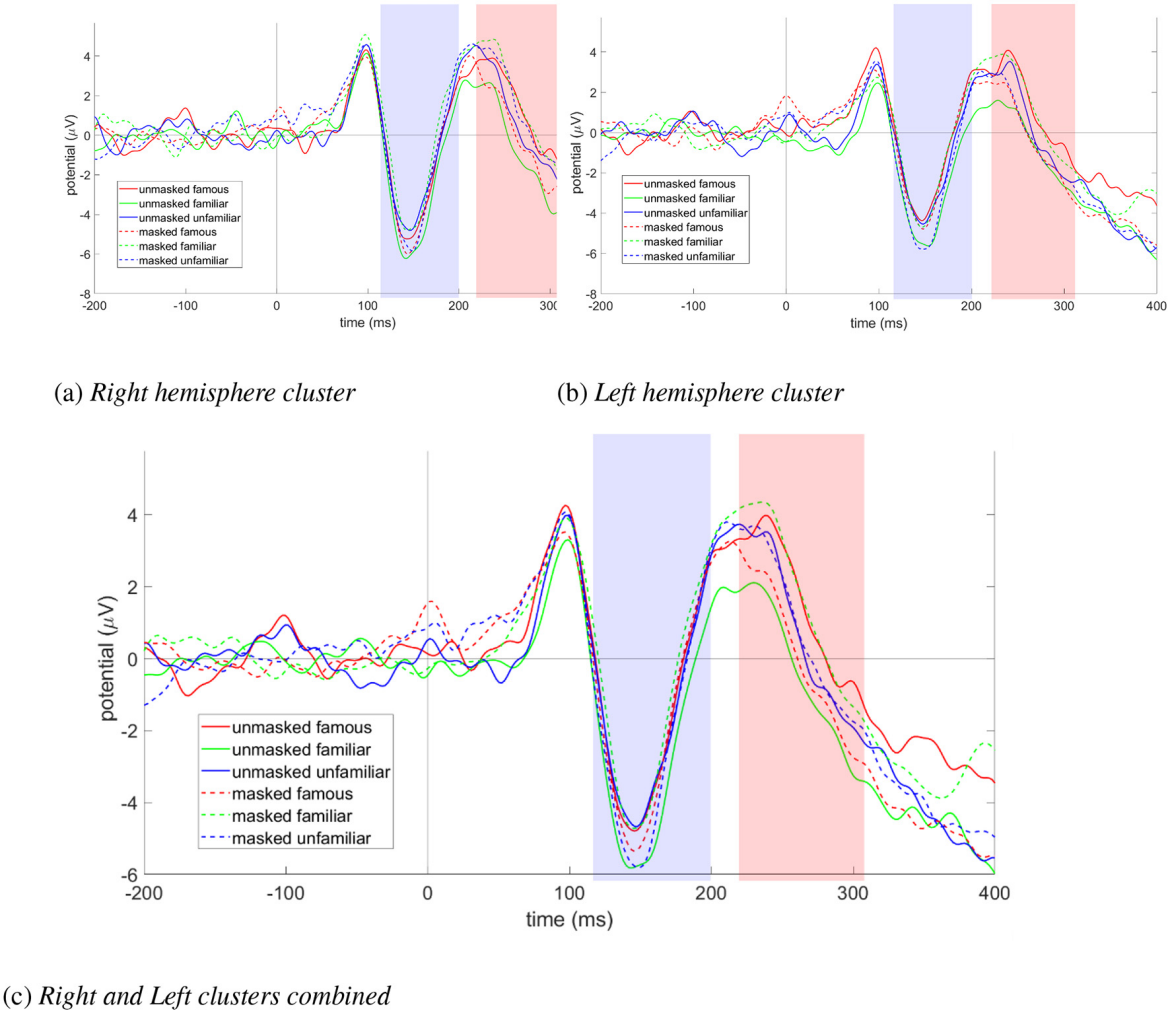
ERP plots of electrode clusters for N170 and N250 ERP components (blue band: N170, red band: N250), grand averaged over all participants (*N* = 18), showing **(a)** right Hemisphere electrode cluster (TP8, P8, P10, PO4, PO8), **(b)** left Hemisphere electrode cluster (TP7, P7, P9, PO3, PO7), and **(c)** right and Left electrode clusters combined (TP7, TP8, P7, P8, P9, P10, PO3, PO4, PO7, PO8).

There were no significant main effects of familiarity [F_(2, 34)_ = 0.18, *p* = 0.838, partial η^2^ = 0.01, BF = 0.06] or mask [F_(1, 17)_ = 0.14, *p* = 0.709, partial η^2^ = 0.008, BF = 0.25]. However, there was a significant interaction effect [F_(2, 34)_ = 3.75, *p* = 0.034, partial η^2^ = 0.181, BF = 1.05]. To investigate this significant interaction in the absence of any significant main effects, we ran one-way ANOVAs to test each factor along the levels of the other factor (see Supplementary Tables 15, 16). We found a significant difference in the N170 response between unmasked and masked personally familiar faces [F_(1, 17)_ = 4.86, *p* = 0.042, η^2^ = 0.222, BF = 2.27] (Figure 9). Specifically, unmasked personally familiar faces elicited a more negative N170 amplitude than masked personally familiar faces. Additionally, the role of the hemisphere (left and right) in this statistically significant difference in the N170 response between unmasked and masked personally familiar faces was tested using a two-way repeated measures ANOVA framework (see Supplementary Table 17), and we found a significant main effect of mask [F_(1, 17)_ = 4.75, *p* = 0.044, partial η^2^ = 0.218, BF = 2.17] but there was no significant main effect of hemisphere [F_(1, 17)_ = 0.07, *p* = 0.797, partial η^2^ = 0.004, BF = 0.25]. The interaction effect was also found to be non-significant (F_(1, 17)_ = 0.001, *p* = 0.983, partial η^2^ = 0.001, BF = 0.24).

#### N250

There were no significant main effects of familiarity [F_(2, 34)_ = 0.25, *p* = 0.777, partial η^2^ = 0.015, BF = 0.04] or mask [F_(1, 17)_ = 0.84, *p* = 0.371, partial η^2^ = 0.047, BF = 0.36]. However, there was a significant interaction effect [F_(2, 34)_ = 7.34, *p* = 0.002, partial η^2^ = 0.302, BF = 17.76]. To investigate this significant interaction in the absence of any main effects, we ran one-way ANOVAs to test each factor along the levels of the other factor (see Supplementary Tables 18, 19). We found significant differences in N250 amplitude between masked and unmasked personally familiar faces $\left[{\text{F}}_{\left(1,17\right)})=6.55,p=0.02,\eta^{2}=0.278,\text{BF}=4.43\right]$ as well as between masked and unmasked famous faces [F_(1, 17)_ = 4.58, *p* = 0.047, η^2^ = 0.212, BF = 2.02] (Figure 9). Specifically, the N 250 response was more negative for unmasked personally familiar faces than for masked personally familiar faces. On the other hand, the N250 response was more negative for masked famous faces than for unmasked famous faces. Additionally, the role of hemisphere (left and right) was analyzed for the above two significant differences (see Supplementary Tables 20, 21).

The two-way repeated measures ANOVA, with mask and hemisphere as factors, on the N250 response for personally familiar faces resulted in a significant main effect of mask [F_(1, 17)_ = 6.55, *p* = 0.02, partial η^2^ = 0.278, BF = 4.43], but there was no significant main effect of hemisphere [F_(1, 17)_ = 0.82, *p* = 0.378, partial η^2^ = 0.046, BF = 0.36]. The interaction effect was also non-significant [F_(1, 17)_ = 0.16, *p* = 0.697, partial η^2^ = 0.009, BF = 0.26].

Similarly, a two-way repeated measures ANOVA, with mask and hemisphere as factors, on the N250 response for famous faces resulted in a significant main effect of mask [F_(1, 17)_ = 4.58, *p* = 0.047, partial η^2^ = 0.212, BF = 2.02] but there was no significant effect of hemisphere [F_(1, 17)_ = 0.07, *p* = 0.797, partial η^2^ = 0.004, BF = 0.24]. The interaction effect was non-significant [F_(1, 17)_ = 0.007, *p* = 0.935, partial η^2^ = 0.001, BF = 0.24].

Importantly, the direction of the effect of face masks on the N250 component was different for personally familiar and famous faces. The unmasked N250 was significantly greater than the masked N250 amplitude for the personally familiar condition. In contrast, the masked N250 was significantly greater than the unmasked N250 amplitude for the famous face condition. The implication of this result is discussed below.

#### ERP results summary

The ERP results showed no effect of familiarity and mask on very early neural processing (P100, 100 ms post-stimulus onset), but a significant difference was found at 170 ms only between unmasked and masked personally familiar faces: the N170 was significantly more negative for unmasked as opposed to masked personally familiar faces. Unfamiliar faces did not elicit any differences in any of the three tested ERPs between their unmasked and masked counterparts. Although, the difference between unmasked and masked unfamiliar faces did not reach significance, it is clear from Figures 8, 9 that masked N170 was more negative than unmasked N170 for unfamiliar faces. This finding is in line with that reported by Prete et al. (2022), where they report a larger N170 for masked faces. Importantly, their study used faces from a pre-existing dataset that were likely unfamiliar to the participants. It is also important to note that they report this effect was stronger in participants with low exposure to masked faces, indicating a general exposure effect that our study specifically investigates.

Additionally, both famous and personally familiar face recognition differed between masked and unmasked conditions in the N250 component; however, the nature of the differences was not the same for personally familiar and famous faces. Specifically, the N250 component was more negative for unmasked vs masked personally familiar faces, whereas it was more negative for masked vs unmasked famous faces. Crucially, this motif of masked ERP being larger than the corresponding unmasked ERP observed in famous face N250 is similar to the unfamiliar face N170 (observed in our study and also reported in Prete et al., 2022), indicating a similar processing difficulty for masked famous faces as for masked unfamiliar faces, albeit emerging later (but also visible in the famous face N170 component, see Figure 9). This is in contrast to the trend observed for personally familiar faces: unlike famous and unfamiliar faces, unmasked N170 for personally familiar faces is more negative than masked N170. This is clear evidence for different underlying mechanisms for the processing of masked personally familiar faces as compared to masked famous and unfamiliar faces.

The neural findings in corroboration with the behavioral results point toward a possible mechanism for additional processing effort required for the recognition of masked faces that are not regularly encountered in one's day-to-day lives (Civile et al., 2012; Colombatto and McCarthy, 2017). An increased N170 for masked than unmasked famous and unfamiliar faces, along with a higher RT for masked famous and masked unfamiliar faces (as compared to a lower masked vs unmasked N170 as well as a lower masked RT for personally familiar faces) is evidence for this additional processing required to identify faces that one is not regularly exposed to in the masked condition. Interestingly, there is no N250 difference between unmasked and masked faces for the unfamiliar category, whereas the higher masked vs unmasked ERP for famous faces sustains even in the N250 ERP component. The N250 component is correlated to the processing of previously known vs unknown faces; thus, this additional processing of masked famous faces sustains much later compared to the processing of masked novel faces.

###  MVPA results

Following channel selection and classification with a linear SVM, the resulting classification accuracies (AUCs) are presented in Supplementary Figure 4. For the classification between masked and unmasked conditions, personally familiar and famous faces show significantly better than chance AUC [Familiar: mean AUC = 54.08, SD = 7.25, t_(17)_ = 2.39, *p* = 0.014, BF = 4.35; Famous: mean AUC = 52.70, SD = 6.4, t_(17)_ = 1.79, *p* = 0.045, BF = 1.71]. Though unfamiliar faces show more than chance AUC, it is not statistically significant [mean AUC = 51.57, SD = 6.26, t_(17)_ = 1.06, *p* = 0.151, BF = 0.66]. Consistent with the behavioral performance accuracy and sensitivity measures, as well as the differences between masked and unmasked N170 and N250 ERP findings, the MVPA results also indicate that participants are better at distinguishing between masked and unmasked faces in the personally familiar and famous categories compared to the unfamiliar category.

## Discussion

Face processing deserves a special place in our life in terms of the social information faces provide, such as age, gender, emotion, identity, among others. However, the advent of the COVID-19 pandemic and the use of face masks have severely affected the way we socially interact with each other. In this study, we explored the effect of mask and familiarity on face recognition using a 2-back task and analyzed the related neural correlates.

###  Effect of familiarity and face mask on face recognition

Face masks used generously during the pandemic have significantly affected our ability to process and recognize faces. Previous behavioral studies in the past couple of years have shown that face masks had a detrimental effect on face processing and recognition (Carragher and Hancock, 2020; Prete et al., 2022; Noyes et al., 2021; Carbon, 2020; Gori et al., 2021; Zochowska et al., 2022; Kollenda and de Haas, 2022). Effect of face masks has also been evident during emotion recognition (Gori et al., 2021; Carbon, 2020; Carbon et al., 2022). Our study shows that in the unmasked condition, recognition was best for personally familiar faces, followed by famous faces while participants performed the worst when encountering an unfamiliar face (Figure 4). This is in concurrence with familiar face recognition literature (Ramon and Gobbini, 2018; Carbon, 2008). In the masked condition, performance decreased for all categories following the same trend shown in recent studies (Carragher and Hancock, 2020; Zochowska et al., 2022; Noyes et al., 2021; Prete et al., 2022), however, the masking effect was most prominent for famous faces (15.95% decrease in performance accuracy in masked compared to unmasked famous faces) followed by personally familiar faces (10.27% decrease in performance accuracy in masked compared to unmasked personally familiar faces) (Figure 4). There was no significant effect of face masks on performance accuracy for unfamiliar faces. The results are consistent with our reaction time (RT) analyses as well. While there was no significant difference in RT between unmasked and masked personally familiar faces, famous and unfamiliar faces showed increased RT for the respective masked condition. The unfamiliar faces also showed the most false positive response compared to personally familiar and famous faces.

Although our results show only a small and non-significant detrimental effect of face masks on the performance accuracy of unfamiliar face recognition, it is likely owing to the inherent difficulty in remembering unknown faces and matching them to previously detected faces in a face memory task. The large number of false positives that were observed for unfamiliar faces (both with and without masks) can be cited to support our hypothesis. It is also important to note that masked unfamiliar faces required a significantly larger RT to produce a similar performance accuracy as their unmasked counterpart. Hence, irrespective of masks, unfamiliar face recognition using visual memory is challenging and requires a longer response time when unfamiliar faces are masked. On the other hand, face masks produce a significant detrimental effect on the performance accuracy for famous and personally familiar faces. Crucially, this reduction in performance is higher for famous than for personally familiar faces. Further, masked famous faces required a significantly larger RT, while there was no statistical difference in the RT for unmasked and masked personally familiar faces. In sum, our results demonstrate that people remember personally familiar faces better, even when they are wearing face masks, alluding to the role of visual experience, while the impairment due to the mask is more prominent for famous faces.

Additionally, using signal detection theory analyses, we show sensitivity to have a similar trend as behavioral performance accuracy, in that observers showed higher sensitivity to unmasked famous and personally familiar faces than their masked counterparts, while inclusion of a mask did not affect the already low sensitivity (and accuracy) for unfamiliar faces. Also, participants showed the highest sensitivity to (and accuracy for) personally familiar faces, across masked and unmasked conditions. Finally, participants were more conservative (higher criterion) in their judgments of famous faces than of both personally familiar and unfamiliar faces. Importantly, face masks did not result in a criterion shift for any of the three familiarity categories. Together, our results show a significant effect of both familiarity and face mask on face recognition and demonstrate a detrimental effect of face mask on face recognition that is different among famous, personally familiar, and unfamiliar faces. We discuss the effect of face masks on famous, personally familiar, and unfamiliar face recognition separately in the following sections.

#### Effect of face mask on famous and unfamiliar face recognition

Most recent face familiarity studies using face masks have used famous faces (Carragher and Hancock, 2020; Noyes et al., 2021) and face matching tasks. Our behavioral results show that the face masks impacted famous face recognition more strongly than personally familiar faces. Recent studies (Kollenda and de Haas, 2022; Carlaw et al., 2022) show an effect of familiarity on masked face recognition, which is in line with our results. A previous study (Noyes et al., 2021) also found deterioration in performance for unfamiliar faces compared to familiar face matching in cross-experimental comparisons. In Carragher and Hancock (2020), the authors interpreted the insignificant effect of familiarity as inconsequential because of different baseline performances for the two conditions. In the experiments citing no effect of familiarity in the masked condition, the task was relatively simple with Carragher and Hancock (2020) using a face matching task where both images were shown simultaneously. However, in studies investigating the face familiarity effect using face masks (including the current study), the task was more challenging. We use a 2-back test while Carlaw et al. (2022) uses a recognition without identification paradigm, and in Kollenda and de Haas (2022), short-term memory for famous and unfamiliar identities in masked conditions was used. Participants identify the two faces within the same trial in matching tasks, whereas, in face memory tasks, including ours, the participants use a stored representation of faces shown previously to compare with the test image. This difference in paradigm might potentially contribute to the difference in results, wherein less challenging face recognition tasks do not show any effect of familiarity when using face masks. The face recognition performance using a “within” design paradigm could contribute to the discrepancy in results from a memory task that uses a “between” design paradigm (Fysh, 2018).

###  Role of visual experience for personally familiar faces

One of the many unprecedented changes that the COVID-19 pandemic enforced on us is the use of face masks, and recent studies have demonstrated that a general deterioration in face processing and recognition can be attributed to the use of face masks (Carragher and Hancock, 2020; Prete et al., 2022; Carbon, 2020; Noyes et al., 2021). However, whether the effect of these forced exposures to masked faces in daily life has contributed to a significant change in face perception and recognition remains relatively unknown. Two longitudinal studies (Carbon et al., 2022; Freud et al., 2022) have failed to show any improvement in performance and show persistent deficits in recognizing masked faces. General exposure to masked faces was not shown to reduce the detrimental effect of face masks [but see Prete et al. (2022)]. Thus, these results point to the mature face processing system not being amenable to changes following prolonged exposure to masks. However, one study (Barrick et al., 2021) demonstrated the effect of visual experience on how people process emotional faces by showing that with prolonged exposure to face masks, participants used more global visual cues for emotion processing. In a separate study (Carragher et al., 2022), it was shown that with diagnostic training, it was possible to improve face processing in the presence of face masks. In the current study, we show evidence of visual experience with face masks and exposure to masked faces in face recognition ability for personally familiar faces when compared with famous faces. Our results show that the difference in performance for personally familiar faces with and without face masks was much lower when compared with the difference in famous faces (Figure 4). This conclusion is also validated by reaction time analysis (Figure 5) where there was no significant difference between masked and unmasked personally familiar face recognition. On the other hand, participants needed longer to process masked famous faces (compared to unmasked famous faces as well as masked personally familiar faces), and yet performed worse in this condition.

Our participants residing on an isolated campus were forced to encounter personally familiar faces for at least 6 months before the experiments were conducted. Thus, our participants, unlike the participants in the longitudinal studies, were not only used to seeing masked faces, but they were forced to view masked faces of the stimuli in real life for a prolonged period prior to the experiment. Real-life interaction with masked personally familiar faces possibly has a role to play in relatively improved performance under masked condition thus alluding to the role of visual experience. Although it can be argued that the enhanced performance for personally familiar faces (with and without face masks) can be attributed simply to the superior performance of personally familiar faces compared to famous faces as evidenced in face familiarity studies (Ramon and Gobbini, 2018; Carbon, 2008), however, the neural results show a different trend for personally familiar faces with masks. The neural correlates of masked vs unmasked personally familiar faces also differ significantly from famous faces, further pointing to the possible role of visual experience. Future studies using a large sample size of personally familiar faces could perhaps clarify the role of the visual experience of face masks on face recognition capability and also shed light on whether the effect of visual experience is sustained for a longer period.

###  Neural correlates of face familiarity and face masks

In the current study, we explore the effect of face masks on famous, personally familiar, and unfamiliar face processing along with their underlying neural correlates. We have analyzed three related ERP components (P100, N170, and N250) and shown that significant differences exist between masked and unmasked personally familiar face recognition in both N170 and N250. Additionally, MVPA results support our behavioral and ERP findings in that masked and unmasked faces were easier to distinguish between for personally familiar and famous individuals, compared to completely unknown (unfamiliar) faces. The effect of face masks was evident for famous faces in only N250, suggesting that the masking effect for personally familiar faces starts as early as 170 ms while for famous faces, the onset is delayed. Crucially, the direction of the difference is opposite between personally familiar and famous faces. Famous faces showed a significantly larger N250 (as well as a larger N170, although not reaching statistical significance), for masked compared to unmasked faces. This effect is reminiscent of the larger N170 for masked (unfamiliar) faces reported by Prete et al. (2022). The opposite was observed for personally familiar faces: masked personally familiar faces elicited a significantly smaller N170 compared to their unmasked counterpart.

There have been a few neural studies (Zochowska et al., 2022; Prete et al., 2022; Proverbio et al., 2023) investigating the effect of face masks out of which one study (Prete et al., 2022) explored the effect of face masks on emotion processing and found N170 and P2 were affected by face masks. This study used unfamiliar faces only. The other study (Zochowska et al., 2022), which is relevant to our work, uses self-image, personally familiar, and unfamiliar images to explore the effect of face masks. Our study, however, shows different results from Zochowska et al. (2022): we find the familiarity effect using face masks, and it is more prominent for personally familiar faces starting from 170 ms onward. As suggested previously, the difference in results can possibly be attributed to the difference in experimental task since our study uses a 2-back paradigm, which is a more challenging task using working memory than a simple detection task used in Zochowska et al. (2022).

A previous study has shown P100 to be a neural correlate of low-level feature encoding of visual cues as opposed to being specific to face perception (Rossion and Caharel, 2011). Another study (Desjardins and Segalowitz, 2013) provides evidence for the unreliability of P100 as a measure of face-specific neural activity. Keeping with this, our study did not find any differences between any of the experimental conditions in the P100 component. This finding, and the fact that we had controlled for low-level features as described in the Stimuli Preparation section, further highlights that our stimuli did not differ in the low-level visual features either between unmasked and masked conditions or among the three familiarity conditions, and any difference in the behavioral and neural measures cannot be attributed to low-level stimuli differences but rather to higher-level face-specific perception and recognition processes.

Whether N170 plays a role in face familiarity is a pending issue in face recognition, and there is evidence supporting both views (see Ramon and Gobbini, 2018 for a review). Since a majority of studies report the consistent effect of familiarity in the later ERP components like N250 (Schweinberger et al., 2002; Wiese et al., 2019, 2021; Tanaka et al., 2006; Bentin and Deouell, 2000; Eimer, 2011), it is generally assumed that N170 reflects the perceptual awareness stage in face recognition akin to “structural encoding” stage in Bruce and Young's standard model of face recognition (Bruce and Young, 1986), and the later ERP components play a more prominent role in face identification using stored memory representations (Bentin and Deouell, 2000; Eimer, 2000b,a, 2011). However, there has been evidence in the literature for earlier processing of face familiarity and the effect is more prominent for personally familiar faces (Ramon and Gobbini, 2018; Caharel et al., 2002, 2005, 2006; Caharel and Rossion, 2021; Barragan-Jason et al., 2015; Huang et al., 2017). In a recent review paper (Caharel and Rossion, 2021), the authors studied the role of N170 in several face familiarity studies and concluded that face familiarity enhances the N170 component and the effect is stronger for personally familiar faces. The authors argue that the onset of familiarity with specific face identities starts as early as 150-200 ms in occipito-temporal areas shortly following the emergence of face selectivity, which emerges right before the familiarity effect. This observation is in contrast with the standard neuro-cognitive models, which distinguish between perceptual and memory processing in human face recognition. Our results are consistent with the findings in Caharel and Rossion (2021) and show a significant difference between masked and unmasked faces for personally familiar faces for the N170 component. N170 is visibly enhanced even for unmasked personally familiar faces when compared with other unmasked faces (see Figure 9). However, the difference does not reach significance. The N170 peak is enhanced for the masked condition for both famous and unfamiliar faces, which is consistent with Prete et al. (2022) (which used unfamiliar faces in their study), although the difference is not significant.

Interestingly, and in contrast to previous studies looking at N170 differences between masked and unmasked unfamiliar faces, the N170 peak amplitude decreases in the masked condition for personally familiar faces, and the same trend is observed for the N250 component as well. The difference between masked and unmasked (personally familiar and famous) faces becomes more prominent in the N250 component, and there is a significant difference for both famous and personally familiar faces. Crucially, the N250 is more enhanced for masked famous faces (in contrast to reduced N250 for masked personally familiar faces) and could be attributed to the greater effort required to process the masked (famous) face. This effect is in line with studies reporting other structural manipulations of the face (Civile et al., 2012; Colombatto and McCarthy, 2017). The attribution of the increased N170 for masked compared to unmasked famous and unfamiliar faces, and a significantly higher N250 for masked than unmasked famous faces, to increased processing effort is also corroborated by evidence from behavioral results, such as increased reaction time for masked famous and masked unfamiliar faces as compared to masked personally familiar faces. It is also important to note that the difference between unmasked and masked unfamiliar faces only appears in the N170 component and, unlike the famous and personally familiar faces, do not sustain through later stages of processing reflected by the N250 component. The N250 is assumed to be a reliable marker of face familiarity and has been shown to reflect memory activation (Ramon and Gobbini, 2018). Thus, it is not surprising that familiarity plays a role in distinguishing between masked and unmasked face stimuli. However, the direction of this change in amplitude between unmasked and masked faces is different for personally familiar and famous faces. For personally familiar faces, N250 amplitude decreases in the masked than the unmasked condition, and this trend is the opposite for famous faces. The decrease in amplitude for personally familiar faces is consistent for both N170 and N250, and we speculate that this decrease can be potentially attributed to visual experience with, and exposure to, masked personally familiar faces, in contrast to the opposite trends observed for famous and unfamiliar face categories. Although further exploration is needed to corroborate this possible link, our study provides one of the earliest evidence of cortical alteration for known face recognition mechanisms owing to prolonged exposure to face masks.

Our study also adds to existing understanding of face perception in general. Previous studies with the N170 ERP component have shown that the eyes, by themselves, were enough to elicit an N170 peak in the observer (Towler et al., 2012; Nemrodov et al., 2017). Following their findings, one may hypothesize that face masks do not alter the N170 component, as the eyes still remain visible on a masked face. However, our study shows differences in the elicited N170 component between unmasked and masked counterparts of the same faces. Thus, we show that face masks disrupt face processing by altering one of the most common neural markers of the same, and add more evidence toward holistic face processing theory (Richler and Gauthier, 2014). Further studies should investigate the effect of face masks and face familiarity, in conjunction with exposure to masked faces. Yet, our current results show convincingly that our cortical activity is transformed after exposure to masks for extended periods. Systematic future studies of the transformed neural activity can potentially enhance our current understanding of the face familiarity effect in general.

###  Limitations

Our study focused on true-positive responses in both behavioral and ERP analyses. Due to the task design, a maximum of 15 true-positive trials per participant per condition could be obtained. On average, approximately 10 trials contributed to the behavioral analyses (M = 10.1, SD = 0.7), and slightly more than 9 trials contributed to the ERP analyses after epoch rejection (M = 9.3, SD = 0.6). The reduced per-participant trial count reflects task difficulty (miss trials), exclusion of faces deemed unfamiliar in post-experiment ratings, and standard EEG artifact rejection procedures (see Materials and Methods). A lower trial count may increase susceptibility to noise and reduce signal stability at the individual level; thus, subtle neural effects should be interpreted cautiously. The relatively modest participant sample and limited stimulus database may also constrain statistical power, particularly for EEG analyses. Larger cohorts would improve statistical sensitivity and neural signal reliability. Nevertheless, the aggregate number of trials per condition across participants remained substantial (>180 for behavioral analyses: M = 181.33, SD = 13.15; >165 for ERP analyses after rejection: M = 167.33, SD = 11.33). Prior simulation work indicates that reliable ERP estimates can be obtained with comparable or even fewer trials (Boudewyn et al., 2018). Future studies permitting a greater number of trials per participant would further strengthen signal reliability and reduce potential bias.

The use of a 2-back task introduces working memory demands, meaning that observed behavioral and neural effects likely reflect face processing under memory load rather than face recognition in isolation. Although this design captures recognition under cognitively demanding conditions, future studies using tasks with reduced memory requirements would help disentangle perceptual recognition mechanisms from memory-related performance costs.

Finally, stimulus heterogeneity across categories represents an additional limitation. Personally familiar faces were photographed under standardized conditions, whereas famous and unfamiliar faces were obtained from different public sources. Despite normalization procedures (e.g., size and luminance), residual differences in lighting, pose, or image quality may remain. Moreover, the stimulus set comprised faces from a relatively homogeneous ethnic background, limiting generalizability. Future research should employ more controlled and demographically diverse stimulus sets to strengthen external validity.

## Conclusion

The current study explores the effect of face masks and familiarity on face recognition, as well as the underlying neural correlates facilitating this recognition. Our results suggest that face recognition is impeded by face masks, as observed in the decrease in performance accuracy and sensitivity for masked personally familiar and masked famous face detection, and the increase in reaction time for masked famous and masked unfamiliar face detection. Our results further suggest a possible role of N170 and N250 in modulating neural signals under masked conditions, and thus altering neural representation during face recognition.

Together, our behavioral and neural evidence demonstrates that a greater effort is required to process and recognize specific kinds of masked faces–both those that are entirely unfamiliar and those that are familiar in their unmasked form but not regularly seen with a mask (e.g., celebrity faces). While the effect for unfamiliar faces replicates previous findings, the effect for famous faces represents a novel contribution. Importantly, our study is one of the first to present behavioral and neural evidence that this increased effort is absent while processing masked personally familiar faces, potentially owing to extended periods of exposure to, and visual experience with, the faces personally known to the observer in both the masked and unmasked forms. Our study explicates the important but not well-studied distinction between the effects of general exposure to masked faces and visual experience with specific masked faces of personally familiar individuals in one's day-to-day environment.

## Data Availability

The raw and processed behavioral data, processed EEG data, and codes for experiment presentation, analysis, and figure generation are made publicly available on a GitHub repository: https://github.com/srijitakarmakar/MaskedFacePerception.git.
