# Supplementary material for: Visual exposure to masked faces benefits personally familiar but not famous face recognition
Source: Front Psychol. 2026 Apr 17;17:1671509. doi: 10.3389/fpsyg.2026.1671509 (PMC13134374; doi:10.3389/fpsyg.2026.1671509)
Supplement: Supplementary file 1 [file Data_Sheet_1.pdf]

Supplementary Information

Additional Face Stimuli Examples

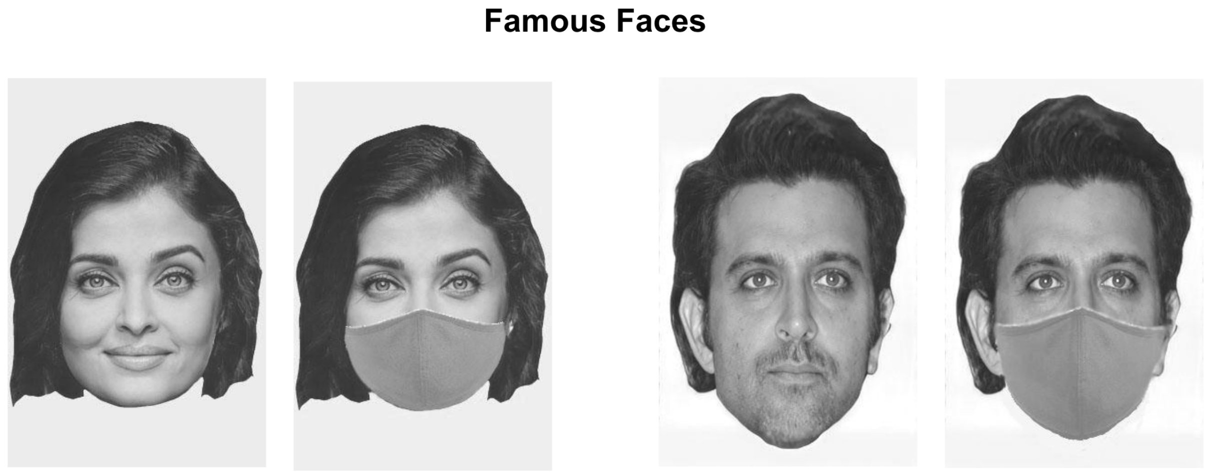

Figure 1: Sample images of Famous faces used in the 2-back task, each shown in both unmasked and masked conditions (all images shown are either under Creative Commons License Attribution.)

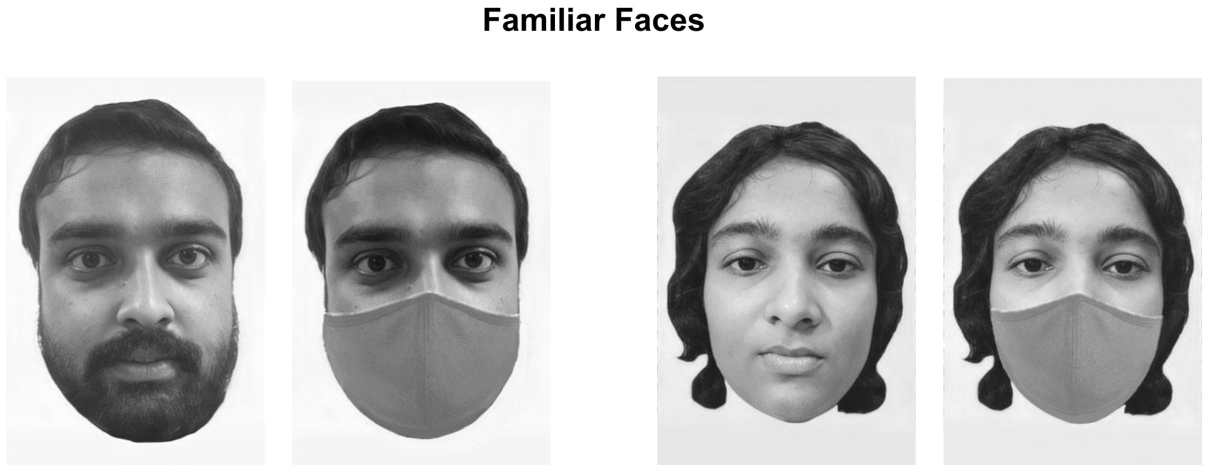

Figure 2: Sample images of Personally Familiar faces used in the 2-back task, each shown in both unmasked and masked conditions (informed consent has been obtained for all images for anonymously displaying them in the public domain.)

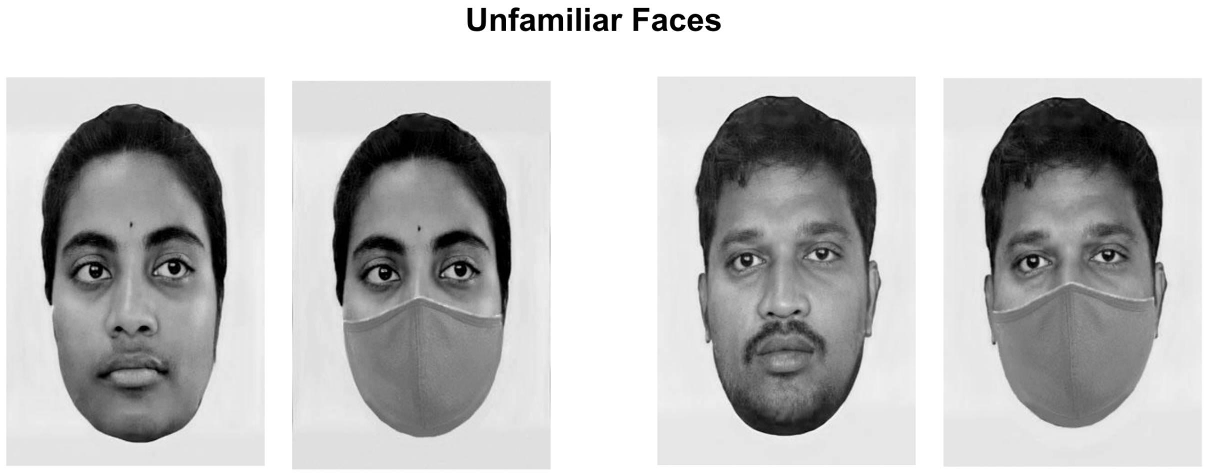

Figure 3: Sample images of Unfamiliar faces used in the 2-back task, each shown in both unmasked and masked conditions (all images shown was made available in a public database [1]).

Behavioral Results

Performance Accuracy (Hit Rate)

We ran a two-way repeated measures ANOVA for the factors of familiarity and mask on behavioral performance accuracy (Table 1) (see Figure 4 in the main text). On finding significant main effects of both familiarity and mask, we followed up the ANOVA with Tukey's HSD post-hoc tests for multiple comparisons (Table 2):

**Dependent Variable: Performance Accuracy**				
**Factor**	**F statistic**	***p*-value**	**partial η^2**	**BF**
Familiarity	10.07	<0.001	0.223	2.63
Mask	12.44	0.001	0.262	39.74
Interaction	2.12	0.127	0.057	0.08

Table 1: Two-way repeated measures ANOVA on performance accuracy

Dependent Variable: Performance Accuracy			
**Group 1**	**Group 2**	***p*-value**	**95% C. I.**
Unmasked Famous	Masked Famous	<0.001	[0.04, 0.15]
Unmasked Familiar	Masked Familiar	0.02	[0.01, 0.14]
Unmasked Unfamiliar	Masked Unfamiliar	0.59	[-0.05, 0.08]
Unmasked Famous	Unmasked Familiar	0.02	[-0.16, -0.01]
Unmasked Famous	Unmasked Unfamiliar	0.48	[-0.04, 0.13]
Unmasked Familiar	Unmasked Unfamiliar	0.004	[0.04, 0.22]
Masked Famous	Masked Familiar	0.003	[-0.19, -0.04]
Masked Famous	Masked Unfamiliar	0.4	[-0.12, 0.04]
Masked Familiar	Masked Unfamiliar	0.08	[-0.01, 0.15]

Table 2: Result of post-hoc Tukey's test on Performance Accuracy

We also ran a two-way repeated measures ANOVA on the data from only those 25 participants for whom EEG data was recorded simultaneously while they participated in the behavioral task. The overall trends of the main effects and interaction effect remain the same as those obtained from the data of the complete sample of 41 participants:

Dependent Variable: Performance Accuracy				
**Factor**	**F statistic**	***p*-value**	**partial η^2**	**BF**
Familiarity	2.86	0.068	0.115	0.2
Mask	6.60	0.018	0.231	4
Interaction	1.28	0.287	0.055	0.05

Table 3: Two-way repeated measures ANOVA on performance accuracy for the subset of participants for whom EEG data was simultaneously recorded

Reaction Time

We ran a two-way repeated measures ANOVA for the factors of familiarity and mask on reaction time (Table 4) (see Figure 5 in the main text). On finding significant main effects of both familiarity and mask, we followed up the ANOVA with Tukey's HSD post-hoc tests for multiple comparisons (Table 5):

Dependent Variable: Reaction Time				
**Factor**	**F statistic**	***p*-value**	**partial η^2**	**BF**
Familiarity	12.62	<0.001	0.265	7.09
Mask	10.73	0.002	0.235	20.52
Interaction	2.16	0.123	0.058	0.08

Table 4: Two-way repeated measures ANOVA on reaction time

Dependent Variable: Reaction Time			
**Group 1**	**Group 2**	***p*-value**	**95% C. I.**
Unmasked Famous	Masked Famous	<0.001	[-0.09, -0.03]
Unmasked Familiar	Masked Familiar	0.33	[-0.05, 0.02]
Unmasked Unfamiliar	Masked Unfamiliar	0.01	[-0.07, -0.01]
Unmasked Famous	Unmasked Familiar	1	[-0.03, 0.04]
Unmasked Famous	Unmasked Unfamiliar	0.01	[-0.09, -0.01]
Unmasked Familiar	Unmasked Unfamiliar	0.003	[-0.09, -0.02]
Masked Famous	Masked Familiar	0.04	[0.002, 0.08]
Masked Famous	Masked Unfamiliar	0.16	[-0.07, 0.01]
Masked Familiar	Masked Unfamiliar	<0.001	[-0.11, -0.04]

Table 5: Result of post-hoc Tukey's test on Reaction Time

We also ran a two-way repeated measures ANOVA on the data from only those 25 participants for whom EEG data was recorded simultaneously while they participated in the behavioral task. The overall trends of the main effects and interaction effect remain the same as those obtained from the data of the complete sample of 41 participants:

Dependent Variable: Reaction Time				
**Factor**	**F statistic**	***p*-value**	**partial η^2**	**BF**
Familiarity	4.62	0.015	0.174	1.34
Mask	8.77	0.007	0.285	10.2
Interaction	0.8	0.455	0.036	0.03

Table 6: Two-way repeated measures ANOVA on Reaction Time for the subset of participants for whom EEG data was simultaneously recorded

Proportion of False-Positive Responses

We ran a two-way repeated measures ANOVA for the factors of familiarity and mask on the proportion of false-positive responses (Table 7) (see Figure 6 in the main text). On

finding significant main effects of both familiarity and mask, we followed up the ANOVA with Tukey's HSD post-hoc tests for multiple comparisons (Table 8):

**Dependent Variable: False-Positive Response Proportion**				
**Factor**	**F statistic**	***p*-value**	**partial η^2**	**BF**
Familiarity	93.77	<0.001	0.728	4.24×10^8
Mask	32.98	<0.001	0.485	2.58×10^4
Interaction	0.41	0.662	0.011	0.04

Table 7: Two-way repeated measures ANOVA on proportion of false-positive responses

**Dependent Variable: False-Positive Response Proportion**			
**Group 1**	**Group 2**	***p*-value**	**95% C. I.**
Unmasked Famous	Masked Famous	<0.0001	[-0.02, -0.01]
Unmasked Familiar	Masked Familiar	<0.001	[-0.03, -0.01]
Unmasked Unfamiliar	Masked Unfamiliar	0.006	[-0.03, -0.01]
Unmasked Famous	Unmasked Unfamiliar	<0.001	[-0.06, -0.04]
Unmasked Familiar	Unmasked Unfamiliar	<0.0001	[-0.05, -0.03]
Masked Famous	Masked Unfamiliar	<0.001	[-0.07, -0.04]
Masked Familiar	Masked Unfamiliar	<0.001	[-0.06, -0.03]

Table 8: Result of post-hoc Tukey's test on proportion of false-positive responses

We also ran a two-way repeated measures ANOVA on the data from only those 25 participants for whom EEG data was recorded simultaneously while they participated in the behavioral task. The overall trends of the main effects and interaction effect remain the same as those obtained from the data of the complete sample of 41 participants:

**Dependent Variable: False-Positive Response Proportion**				
**Factor**	**F statistic**	***p*-value**	**partial η^2**	**BF**
Familiarity	58.93	<0.001	0.728	1.45×10^6
Mask	19.32	<0.001	0.467	4.6×10^2
Interaction	1.37	0.264	0.056	0.055

Table 9: Two-way repeated measures ANOVA on proportion of false-positive responses for the subset of participants for whom EEG data was simultaneously recorded

Signal Detection Theoretic Analysis: Sensitivity

We first report the mean sensitivity for each experimental condition. Unmasked and masked famous faces elicited a sensitivity of 3.363 ± .154 and 2.615 ± .169, respectively.

Unmasked and masked personally familiar faces showed a sensitivity of 3.761 ± .189 and 2.750 ± .147, respectively. Finally, unmasked and masked unfamiliar faces showed a sensitivity of 2.164 ± .147 and 1.882 ± .093, respectively.

We ran a two-way repeated measures ANOVA for the factors of familiarity and mask on sensitivity (d') (Table 10). On finding significant main effects of both familiarity and mask, as well as a significant interaction effect, we followed up the ANOVA with Tukey's HSD post-hoc tests for multiple comparisons:

**Dependent Variable: Sensitivity**				
**Factor**	**F statistic**	***p*-value**	**partial η^2**	**BF**
Familiarity	44.78	<0.001	0.561	7.67×10^4
Mask	37.98	<0.001	0.52	9.25×10^4
Interaction	4.94	0.01	0.124	0.3

Table 10: Two-way repeated measures ANOVA on sensitivity (d')

As there is a significant interaction effect, main effects are no longer interpretable. We find the following from post-hoc tests on the interaction effect: there is a significant effect of the 'Mask' factor (Bonferroni corrected $p < .001$) on the 'famous' and 'personally familiar' levels of the 'Familiarity' factor, but not on the 'unfamiliar' level of the 'Familiarity' factor.

There was also a significant effect of 'Familiarity' (Bonferroni corrected $p<.001$) of both 'unmasked' and 'masked' levels of the 'Mask' factor.

There were significant differences (Bonferroni corrected $p<0.001$) between unmasked familiar and unfamiliar, unmasked famous and unfamiliar, masked familiar and unfamiliar, masked famous and unfamiliar, unmasked and masked familiar, and unmasked and masked famous.

No significant difference in sensitivity was found between unmasked and masked unfamiliar, or between famous and familiar, within either unmasked or masked conditions.

Signal Detection Theoretic Analysis: Criterion

We first report the mean criterion for each experimental condition. Unmasked and masked famous faces showed a criterion of .915 ± .072 and .905 ± .064, respectively. Unmasked

and masked personally familiar faces showed a criterion of .621 ± .094 and .592 ± .078, respectively. Finally, unmasked and masked unfamiliar faces showed a criterion of .440 ± .076 and .475 ± .061, respectively.

We ran a two-way repeated measures ANOVA for the factors of familiarity and mask on criterion (d') (Table 11). On finding significant main effects of both familiarity and mask, as well as a significant interaction effect, we followed up the ANOVA with Tukey's HSD post-hoc tests for multiple comparisons:

**Dependent Variable: Sensitivity**				
**Factor**	**F statistic**	***p*-value**	**partial η^2**	**BF**
Familiarity	24.12	<0.001	0.41	3.48×10^2
Mask	.0008	0.98	0	0.17
Interaction	.22	0.78	0.006	0.03

Table 11: Two-way repeated measures ANOVA on criterion (λ)

We ran Tukey's post-hoc tests to find which levels of the 'Familiarity' factor showed significant differences between the 'unmasked' and 'masked' levels of the 'Mask' factor. The criterion is significantly different between unmasked famous and unmasked familiar conditions (Bonferroni corrected p=.002), between unmasked famous and unmasked unfamiliar (Bonferroni corrected p<.001), masked famous and masked familiar (Bonferroni corrected p=.001), and masked famous and masked unfamiliar (Bonferroni corrected p<.001).

The criterion was not significantly different between familiar and unfamiliar conditions, either within the unmasked or the masked types. For all levels of the 'Familiarity' factor, there were no significant differences in the criterion between the unmasked and masked conditions.

Neural Results

P100

Two-way repeated measures ANOVA for the factors of familiarity and mask was carried out for the P100 ERP component:

Dependent Variable: P100 ERP amplitude				
**Factor**	**F statistic**	**p-value**	**partial η^2**	**BF**
Familiarity	1.34	0.276	0.077	0.11
Mask	0.08	0.777	0.005	0.25
Interaction	2.57	0.092	0.139	0.2

Table 12: Two-way repeated measures ANOVA on P100 ERP amplitude

We did not find any significant main effect or interaction effect in the above ANOVA. Thus, we separated the data by hemispheres (left electrode cluster and right electrode cluster), and ran a two-way repeated measures ANOVA for each:

Dependent Variable: P100 ERP amplitude (RH)				
**Factor**	**F statistic**	**p-value**	**partial η^2**	**BF**
Familiarity	0.79	0.465	0.047	0.08
Mask	0.48	0.5	0.029	0.3
Interaction	1.77	0.187	0.099	0.14

Table 13: Two-way repeated measures ANOVA on P100 ERP amplitude, right hemisphere cluster

Dependent Variable: P100 ERP amplitude (LH)				
**Factor**	**F statistic**	**p-value**	**partial η^2**	**BF**
Familiarity	1.33	0.279	0.077	0.11
Mask	0.02	0.9	0.001	0.24
Interaction	2.16	0.132	0.112	0.16

Table 14: Two-way repeated measures ANOVA on P100 ERP amplitude, left hemisphere cluster

N170

First, a two-way repeated measures ANOVA for the factors of familiarity and mask was carried out for the N170 ERP component:

On finding a significant interaction effect and no significant main effects, we ran one-way ANOVAs to test each factor along the levels of the other factor:

Dependent Variable: N170 ERP amplitude				
**Factor**	**F statistic**	***p*-value**	**partial η^2**	**BF**
Familiarity	0.18	0.838	0.01	0.06
Mask	0.14	0.709	0.008	0.25
Interaction	3.75	0.034	0.181	1

Table 15: Two-way repeated measures ANOVA on N170 ERP amplitude

Dependent Variable: N170 ERP amplitude				
**Treatment Conditions**	**F statistic**	***p*-value**	**η^2**	**BF**
Unmasked: Famous, Familiar, Unfamiliar	2.33	0.113	0.121	0.75
Masked: Famous, Familiar, Unfamiliar	2.26	0.12	0.117	0.72
Famous: Unmasked, Masked	1.28	0.273	0.07	0.45
Familiar: Unmasked, Masked	4.86	0.042	0.222	2.27
Unfamiliar: Unmasked, Masked	0.18	0.675	0.011	0.26

Table 16: One-way repeated measures ANOVA on N170 ERP amplitude

Next, we tested the effect of hemisphere (left and right) on the significant differences found in the one-way ANOVAs. In this case, the only significant difference was observed between unmasked and masked personally familiar faces. We investigated this further with two-way ANOVA with "mask" and "hemisphere" as factors:

Dependent Variable: N170 ERP amplitude, Familiar Face				
**Factor**	**F statistic**	***p*-value**	**partial η^2**	**BF**
Mask	4.75	0.044	0.218	2.17
Hemisphere	0.07	0.797	0.004	0.2
Interaction	0.001	0.983	0.001	0.24

Table 17: Two-way repeated measures ANOVA on N170 ERP amplitude for Familiar Face with Mask and Hemisphere as two factors

A significant main effect of mask was found, but no significant difference on hemisphere was noted.

N250

First, a two-way repeated measures ANOVA for the factors of familiarity and mask was carried out for the N250 ERP component:

Dependent Variable: N250 ERP amplitude				
**Factor**	**F statistic**	***p*-value**	**partial η^2**	**BF**
Familiarity	0.25	0.777	0.015	0.04
Mask	0.84	0.371	0.047	0.36
Interaction	7.34	0.002	0.302	17.78

Table 18: Two-way repeated measures ANOVA on N250 ERP amplitude

On finding a significant interaction effect and no significant main effects, we ran one-way ANOVAs to test each factor along the levels of the other factor:

Dependent Variable: N250 ERP amplitude				
**Treatment Conditions**	**F statistic**	***p*-value**	η^2	**BF**
Unmasked: Famous, Familiar, Unfamiliar	3.26	0.051	0.161	1.14
Masked: Famous, Familiar, Unfamiliar	2.78	0.076	0.141	0.92
Famous: Unmasked, Masked	4.58	0.047	0.212	2
Familiar: Unmasked, Masked	6.55	0.02	0.278	4.43
Unfamiliar: Unmasked, Masked	1.3	0.27	0.071	0.46

Table 19: One-way repeated measures ANOVA on N250 ERP amplitude

Next, we tested the effect of hemisphere (left and right) on the significant differences found in the one-way ANOVAs. In this case, the only significant differences were observed between (1) unmasked and masked personally familiar faces and between (2) unmasked and masked famous faces.

Dependent Variable: N250 ERP amplitude, Familiar Face				
**Factor**	**F statistic**	***p*-value**	**partial η^2**	**BF**
Mask	6.55	0.02	0.278	4.43
Hemisphere	0.82	0.378	0.046	0.36
Interaction	0.16	0.697	0.009	0.26

Table 20: Two-way repeated measures ANOVA on N250 ERP amplitude for Familiar Face with Mask and Hemisphere as two factors

Dependent Variable: N250 ERP amplitude, Famous Face				
**Factor**	**F statistic**	***p*-value**	**partial η^2**	**BF**
Mask	4.58	0.047	0.212	2.02
Hemisphere	0.07	0.797	0.004	0.24
Interaction	0.007	0.935	0.001	0.24

Table 21: Two-way repeated measures ANOVA on N250 ERP amplitude for Famous Face with Mask and Hemisphere as two factors

MVPA Results

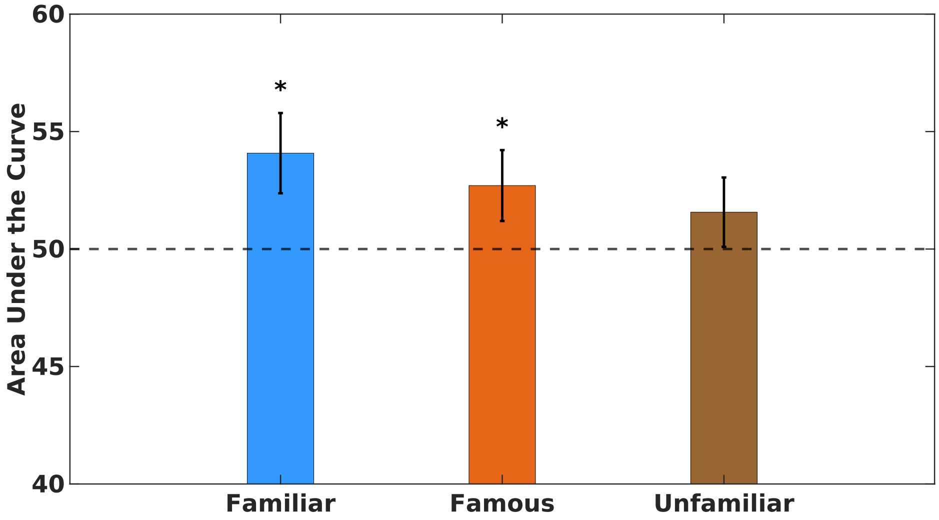

Figure 4: Area Under the Receiver Operator Characteristic Curve (AUC) after selecting EEG channels for all three face familiarity types between masked vs unmasked conditions. Vertical lines indicate the standard error of the mean AUC, and the horizontal dashed line represents the chance AUC. The AUC was significantly better than chance for personally familiar and famous faces, indicated by the * sign on top of the bars.

References

[1] Lazarus Mayaluri, Supratim Gupta, and Nidhi Panda. "An Indian facial database highlighting the Spectacle Problems". In: *IEEE Dataport* (2019). DOI: `https://dx.doi.org/10.21227/ay8v-kj06`.
